# Cryo-EM structure of an activated VIP1 receptor-G protein complex revealed by a NanoBiT tethering strategy

**DOI:** 10.1038/s41467-020-17933-8

**Published:** 2020-08-17

**Authors:** Jia Duan, Dan-dan Shen, X. Edward Zhou, Peng Bi, Qiu-feng Liu, Yang-xia Tan, You-wen Zhuang, Hui-bing Zhang, Pei-yu Xu, Si-Jie Huang, Shan-shan Ma, Xin-heng He, Karsten Melcher, Yan Zhang, H. Eric Xu, Yi Jiang

**Affiliations:** 1 The CAS Key Laboratory of Receptor Research, Shanghai Institute of Materia Medica, Chinese Academy of Sciences, Shanghai, 201203 China; 2 University of Chinese Academy of Sciences, 100049 Beijing, China; 3 Department of Pathology of Sir Run Run Shaw Hospital, Zhejiang University School of Medicine, Hangzhou, 310058 China; 4 Department of Biophysics, Zhejiang University School of Medicine, Hangzhou, 310058 China; 5 Center for Cancer and Cell Biology, Program for Structural Biology, Van Andel Institute, Grand Rapids, MI USA; 6 School of Life Science and Technology, ShanghaiTech University, Shanghai, 201210 China

**Keywords:** G protein-coupled receptors, Cryoelectron microscopy

## Abstract

Vasoactive intestinal polypeptide receptor (VIP1R) is a widely expressed class B G protein-coupled receptor and a drug target for the treatment of neuronal, metabolic, and inflammatory diseases. However, our understanding of its mechanism of action and the potential of drug discovery targeting this receptor is limited by the lack of structural information of VIP1R. Here we report a cryo-electron microscopy structure of human VIP1R bound to PACAP27 and Gs heterotrimer, whose complex assembly is stabilized by a NanoBiT tethering strategy. Comparison with other class B GPCR structures reveals that PACAP27 engages VIP1R with its N-terminus inserting into the ligand binding pocket at the transmembrane bundle of the receptor, which subsequently couples to the G protein in a receptor-specific manner. This structure has provided insights into the molecular basis of PACAP27 binding and VIP receptor activation. The methodology of the NanoBiT tethering may help to provide structural information of unstable complexes.

## Abstract (web summary)

Vasoactive intestinal polypeptide receptor (VIP1R) is a widely expressed class B G protein-coupled receptor and a drug target for the treatment of inflammatory diseases. Here authors report a cryoelectron microscopy structure of human VIP1R bound to PACAP27 and Gs heterotrimer, which provides insights into PACAP27 binding and VIP receptor activation.

## Introduction

Vasoactive intestinal polypeptide receptors, also known as VIP receptors, including VIP1R and VIP2R, belong to the class B1 of G protein-coupled receptors. Upon activating by vasoactive intestinal peptide (VIP), an endogenous, 28-amino acid neuropeptide, a VIP receptor couples to Gs heterotrimer, resulting in the stimulation of adenylyl cyclase. In addition to VIP, VIP receptors also bind to other neuropeptides called pituitary adenylate cyclase-activating peptides (PACAPs) with similar affinity. Two forms of PACAP are known, the 27 amino acid long PACAP27 and the 38 amino acid long PACAP38, of which PACAP27 is a C terminally truncated variant of PACAP38, and shows particularly high homology (~68%) to VIP. The PACAP peptides have been in the spotlight of extensive basic and applied research, and have been linked to for over 40 different pathological conditions with clinical relevance^1^.

VIP1R is widely distributed in the CNS, most abundantly in the cerebral cortex and hippocampus^2,3^, where it plays diverse and important roles with functions in the control of circadian rhythms, learning, memory, anxiety and responses to stress, and brain injury. VIP1R is also expressed in a number of peripheral tissues, including liver, lung, and intestine^2–8^, and in T lymphocytes^9^. The development of drugs acting on VIP receptors may lead to new treatments for sleep disorders, stroke, neurodegenerative disorders, and age-related memory impairment.

Extensive efforts have been made to discover the roles of the VIP1R system and to take advantage of VIP and PACAP analogs in therapeutic applications. Understanding the mechanism of peptide recognition and signal transduction by VIP1R has been aided by insights from several functional data from mutagenesis, photoaffinity labeling^10^, molecular modeling^11,12^, and limited structure information of VIP2R extracellular domain (ECD) (PDB code: 2X57) and VIP peptide^13^. Several of VIP and PACAP peptide analogs have been studied for their potential therapeutic applications^1^. A high-resolution structure of a full-length VIP receptor is needed for both mechanistic research as well as drug discovery targeting this GPCR system.

The resolution revolution of cryo-EM has made a significant impact on GPCR structural biology^14^. The atomic resolution or near-atomic resolution GPCR–G protein complex structures solved by cryo-EM have revealed structural details of ligand recognition and signal transduction by this superfamily of cell surface receptors. Various methods have been developed to improve the stability of GPCR-signal transducer complexes, such as the use of thermo-stabilizing mutations^15^, nanobodies, and antibody fragments^16,17^, to facilitate structural studies. However, poor sample stability remains the bottleneck in structural studies of GPCR complexes. In this work, we have developed a method to stabilize the interaction between VIP1R and the Gs heterotrimer by bringing the two proteins into close proximity through a NanoBiT tethering approach. This method greatly improved the stability and homogeneity of the PACAP27–VIP1R–Gs protein complex, allowing structural determination of human VIP1R in complex with PACAP27 and Gs heterotrimer. We also demonstrate that the NanoBiT tethering method can be applied to other GPCR–G protein complexes.

## Results

### The tethered NanoBiT stabilize GPCR–G protein complexes

NanoBiT system is one of the protein-fragment complementation methods based on split luciferase, which is originally developed to monitor protein-protein interactions^18^. When the NanoBiT is dissected between residues 156 and 157, it can be split into a large component containing 156 amino acid residues named large BiT (LgBiT), and a 13-amino acid peptide called small BiT (SmBiT, Supplementary Fig. 1a). By engineering the sequence of SmBiT, a series of peptides with various equilibrium dissociation constants were created, among which peptide 86 (HiBiT) (VSGWRLFKKIS) has the most potent binding affinity, with five orders of magnitude (~1 nM to ~200 μM) greater than that of the wild-type (WT) peptide 114 (VTGYRLFEEIL) (Supplementary Fig. 1b)^18^. The fragments of the LgBiT and SmBiT are genetically fused to a pair of interacting proteins. The interaction of fusion partners leads to structural complementation of LgBiT with SmBiT, generating a functional NanoBiT enzyme with a detectable luminescent signal.

**Fig. 1:**
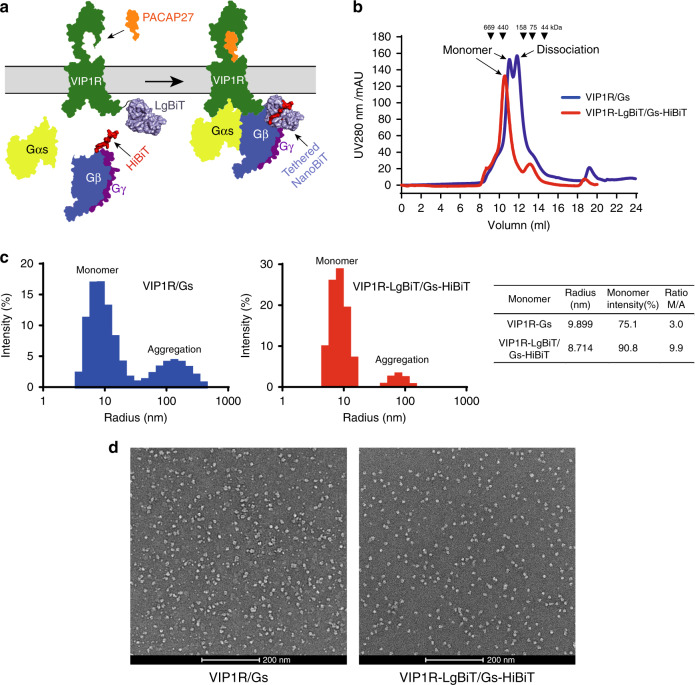
The NanoBiT strategy for stabilization of PACAP27–VIP1R–Gs protein complex. **a** Schematic diagram of the NanoBiT aided assembly of the VIP1R–Gs complex. PACAP27 is colored in orange, VIP1R in green, Gαs in yellow, Gβ in blue, Gγ in purple, LgBiT in light blue, and HiBiT in red. **b** Respective size-exclusion chromatography elution profiles of the VIP1R–Gs and VIP1R-LgBiT-Gs-HiBiT complexes. **c** Dynamic light scattering (DLS) size distribution histograms of VIP1R–Gs and VIP1R-LgBiT-Gs-HiBiT complexes. Values of radius, % intensity of monomer, and ratio of monomer/aggregation (M/A) are listed. **d** Representative negative staining images of the corresponding complexes. The scale bar is 200 nm. Source data are provided as a Source Data file.

Inspired by the complementation principle of NanoBiT, we fused the SmBiT peptide 86 at the C-terminus of the Gβ subunit to bind the LgBiT that was attached to the C-terminus of the truncated receptor (VIP1R 31–437), thus providing an additional linkage to stabilize the interface of helix 8 of VIP1R and the Gβ subunit of the G protein (Fig. 1a, Supplementary Fig. 2; see “Methods”). The flexible C-terminus of VIP1R serves as the natural linker to connect LgBiT. The WT VIP1R and different lengths of C-terminus truncated VIP1R at L437, G424, and K417 were screened for assembly of the complex. When the receptor was truncated to L437, the components can be assembled into the VIP1R(31–437)–VIP1R–Gs complex with an equal proportion, suggesting a better assembly efficacy for the complex. Thus, unless otherwise specified, VIP1R refers to VIP1R(31–437), which is used in structure determination and functional analyses. Compared to WT VIP1R, the truncated receptor exhibited a comparable response to PACAP27-induced activation. The LgBiT fusion to the truncated VIP1R or cotransfection of the truncated receptor with Gβ-HiBiT does not affect PACAP27-induced VIP1R activation (Supplementary Fig. 1c). Combined with Nb35, which is used to stabilize the complex between Gαs and Gβ^19^, the NanoBiT tethering method can enhance the stability of the VIP1R–Gs complex and facilitate the structure study of this GPCR complex.

To investigate the effect of the NanoBiT tethering method on stabilization of the PACAP27–VIP1R–Gs complex, SDS-PAGE analysis, gel filtration chromatography, dynamic light scattering (DLS), and negative staining was performed. The SDS-PAGE analysis showed that all components of the VIP1R–Gs complex were present with the NanoBiT tethering (Supplementary Fig. 1d). Gel filtration chromatography also revealed that the complex with the NanoBiT tethering had a much more uniform distribution than the WT VIP1R–Gs complex, indicating that the NanoBiT tethering method contributed additional stability to the VIP1R–Gs complex (Fig. 1b).

We further used DLS to evaluate complex homogeneity and thermostability. A peak around a radius of ~10 nm corresponds to the monomeric complex of VIP1R–Gs complex, while the peak at ~100 nm represents protein aggregation. Our data show that the NanoBiT tethering improved the monodispersity of the VIP1R complex with a 3.3-fold increase of monomer/aggregation ratio (Fig. 1c). The relatively smaller radius size and narrower radius size distribution also suggest that the NanoBiT tethering complex was more compact and homogeneous than the WT complex, while the protein aggregation onset temperature (*T*_onset_), a marked temperature point indicating protein denaturation and aggregation, remained unchanged (Supplementary Fig. 1e). The negative staining images displayed that particle morphology and integrity of the NanoBiT-tethered complex have been improved relative to the less consistent particles of the WT complex, indicating improved homogeneity and integrity of the NanoBiT-tethered sample (Fig. 1d).

We further investigated whether NanoBiT tethering conferred a similar stabilization effect on other GPCR–G protein complexes. CCR7, a class A GPCR that couples to Gi protein, was chosen as a representative receptor. The NanoBiT tethering method significantly improved the homogeneity of the complex, leading to high homogeneity and integrity of negatively stained complex particles, which is in agreement with its effect on the VIP1R–Gs complex. The NanoBiT tethering also increased the thermostability of the CCR7-Gi complex, as evidenced by an increase of *T*_onset_ by ~10 °C (Supplementary Fig. 3).

Taken together, we developed a strategy to stabilize the GPCR–G protein complex by direct linking of a GPCR with its G protein through NanoBiT protein-fragment complementation. Using this method, we were able to obtain a stable PACAP27–VIP1R–Gs complex for cryo-EM studies.

### Structure determination of VIP1R bound to PACAP27 and Gs

**Fig. 2:**
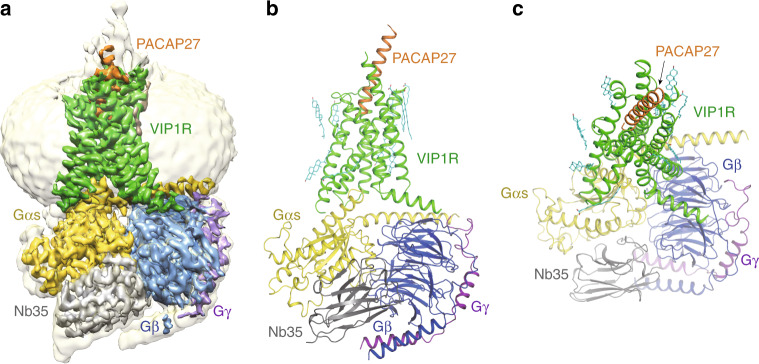
The overall cryo-EM structure of PACAP27–VIP1R–Gs complex. **a** A cut-through view of the cryo-EM map of PACAP27–VIP1R–Gs complex with a disc-shaped micelle. **b** A cartoon representation of the PACAP27–VIP1R–Gs complex. **c** Extracellular view of the PACAP27–VIP1R–Gs complex structure. PACAP27 is colored in orange; VIP1R in green; Gαs Ras-like domain in yellow; Gβ subunit in blue; Gγ subunit in purple; Nb35 in gray; and lipid molecules in cyan.

The structure of the PACAP27–VIP1R–Gs complex was determined from 131,263 particles to a resolution of 3.2 Å (Supplementary Fig. 4 and Supplementary Table 1). The density is clear for the VIP1R TM bundle, the bound peptide PACAP27, the heterotrimeric Gs, and Nb35. Like many other GPCR–G protein complexes, density is missing for the α-helical domain of the Gαs. In addition, the ECD of VIP1R was not resolvable with this limited dataset, perhaps reflecting its highly dynamic and conformationally flexible property when bound to PACAP27. This is consistent with the highly dynamic nature of ECD in class B GPCRs when bound to activating ligands as the ECD structures were not well resolved in most of other active class B GPCR–G protein complexes^20–22^. The complex structure of PACAP27–VIP1R–Gs was built with the recently published PTH1R–Gs complex structure (PDB: 6NBH)^23^ as an initial model. The final structure contains all residues of PACAP27 (residues 1–27), the Gαs Ras-like domain, Gβγ subunits, Nb35, and the VIP1R residues from A129^1.26b^ to Q409^8.64b^ (class B GPCR numbering in superscript^24^) (Fig. 2). The majority of amino acid side chains were well resolved in the final model, which were refined against the EM density map (Supplementary Fig. 5). Thus, the complex structure can provide detailed information on the interface between Gαs and the receptor, as well as the binding interface between PACAP27 and helix bundle core of the receptor.

The TMD of the VIP1R receptor is surrounded by an annular detergent micelle mimicking the natural phospholipid bilayer. Within the micelle, six cholesterol molecules are clearly visible in the cryo-EM density map (Fig. 2), which hydrophobically binds around the helix bundle of the receptor and may contribute to the stability of the receptor-ligand binding^23^.

Interestingly, the density of the NanoBiT is invisible in our structure. We suspect that the NanoBiT can increase the local concentration of Gs heterotrimer near VIP1R, and also make the Gs heterotrimer not easily dissociated from the receptor. Compared with the VIP1R–Gs protein complex, the NanoBiT is relatively flexible because of the existence of the linker between LgBiT and the receptor. The stabilization mode of NanoBiT is different from the antibodies that bind against the antigen directly and can be traced in a clear density map.

### PACAP recognition by VIP1R and PAC1R

**Fig. 3:**
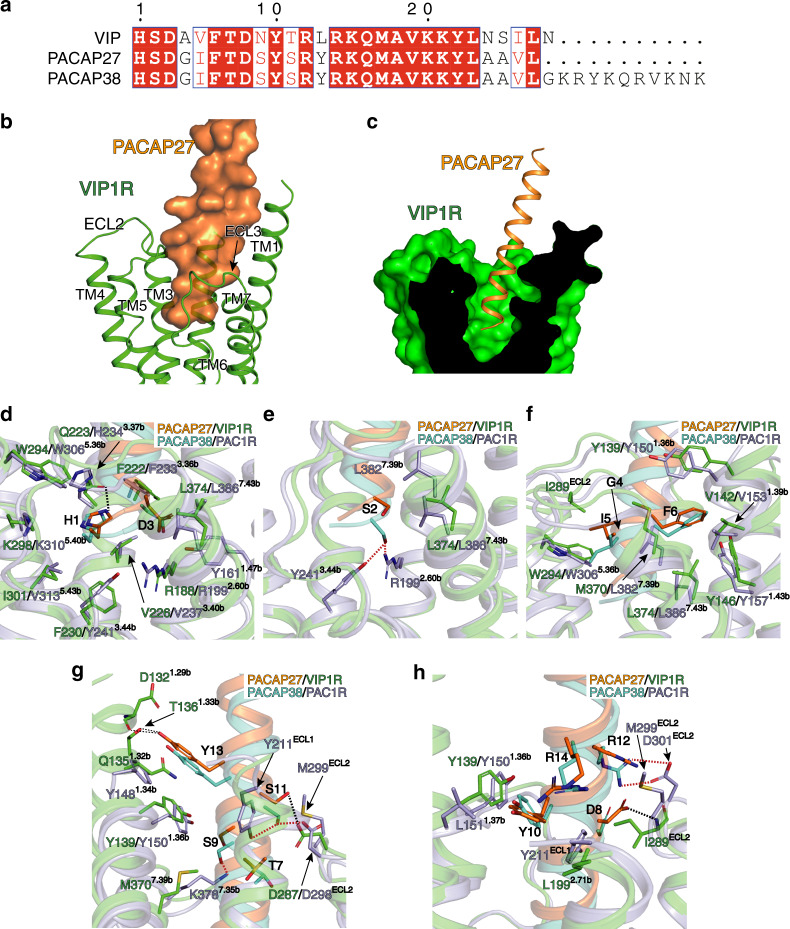
Comparison of the binding mode of PACAPs to VIP1R and PAC1R. **a** Sequence alignment of the VIP1R peptide ligands VIP, PACAP27, and PACAP38. **b** The binding mode of PACAP27 to VIP1R, showing that PACAP27 adopts α-helical conformation and interacts with all TM helices of VIP1R except TM4. **c** The cross-section view of the PACAP27 binding pocket in the TM bundle of VIP1R. Structural comparisons of PACAP binding pockets in VIP1R and PAC1R. Residues interact with peptide amino acids H1 and D3 (**d**), S2 (**e**), G4, I5, and F6 (**f**), as well as amino acids from T7 to R14 (**g**, **h**) are shown as sticks. The hydrogen bonds between PACAP27 and residues of VIP1R are marked as black dotted lines, and the hydrogen bonds between PACAP38 and residues of VIP1R are shown as red dotted lines. PACAP27 is colored in orange, and VIP1R in green. PACAP38 is shown in cyan, and PAC1R (PDB code: 6P9Y) in light blue.

The activated VIP1R complex shows that PACAP27 adopts α-helical conformations and engages a V-shape binding pocket with a prominent open cleft at the extracellular part of the helix bundle. PACAP27 interacts with each of the TM helices except TM4, with the N-terminus of the peptide inserting deeply into the TMD core. ECL2 and ECL3 also mediate the interaction between peptide and receptor (Fig. 3b, c).

Compared to other peptide ligands bound in the pockets of their cognate class B GPCRs^21,23,25–28^, PACAP27 shows different conformations primarily at its C-terminal end and is differently oriented in the ligand binding pocket of the receptor (Supplementary Fig. 6a, b). In contrast, the N-terminal ends of all peptide ligands are well overlapped among different members of class B GPCRs (Supplementary Fig. 6b). The orientation of a peptide ligand in the ligand binding pocket of a class B GPCR is determined by the specific interactions of the N-terminal portion of the peptide ligand with the TMD of the receptor, which keeps the peptide ligand in a specific position in the ligand binding pocket. The TMD peptide-binding pocket of VIP1R is similar to that of PAC1R with pocket volumes of 3261 and 3246 Å^3^, respectively, as these two receptors share peptidic ligand PACAP with similar affinity. Notably, the TMD peptide-binding pocket of VIP1R and PAC1R are smaller than those of class B GPCRs solved to date (Supplementary Fig. 6c and Supplementary Table 2).

A comparison of the structures of PACAP27–VIP1R–Gs with the newly released PACAP38–PAC1R–Gs complex (PDB: 6P9Y)^21^ will help to clarify the recognition mechanism of VIP1R and PAC1R by PACAP peptide. For VIP1R, the first peptide residue H1 not only makes extensive hydrophobic contacts with several nonpolar residues from the ligand pocket (V226^3.40b^, F230^3.44b^, W294^5.36b^, I301^5.43b^, and the backbone of K298^5.40b^) but also forms a hydrogen bond with Q223^3.37b^ (Fig. 3d, Supplementary Table 3). Alanine mutations in Q223^3.37b^ and W294^5.36b^ reduced PACAP27-mediated VIP1R activation, supporting the fact that H1 of the peptide is critical for peptide-induced receptor activation (Supplementary Table 4). For PAC1R, H1 interacts with highly conserved hydrophobic residues with VIP1R. However, the hydrogen bond interacts with residue at 3.37 (H234^3.37^) is absent (Fig. 3d, Supplementary Table 3). Compared to H1, S2 faces a significant different residue environment in these two receptors. S2 additionally hydrogen-bonded with R199^2.60^ and Y241^3.44^ in PAC1R compared to VIP1R, making the PACAP38 inserted deeper into the TMD core of PAC1R (Fig. 3e, Supplementary Table 3). D3 forms hydrogen bond with R188^2.60^, and also hydrophobic contacts with F222^3.36^ and L374^7.43^. These interactions are highly conserved between VIP1R and PAC1R (Fig. 3d, Supplementary Table 3). Mutations of the peptide residue D3 or the receptor residue R188^2.60b^ and F222^3.36^ impaired ligand-induced receptor activation^29^ (Supplementary Table 4). G4, I5, and F6 from the peptide ligand are surrounded by hydrophobic pocket residues of VIP1R from TM1 (Y139^1.36b^, V142^1.39b^, Y146^1.43b^), TM2 (L199^2.71b^), TM5 (W294^5.36b^), and TM7 (M370^7.39b^ and L374^7.43b^), as well as from ECL2 (I289^ECL2^) (Fig. 3f, Supplementary Table 3). Mutations of hydrophobic residues Y146^1.43b^, L199^2.71b^, and W294^5.36b^ to alanines significantly decreased the PACAP27-induced VIP1R activation, indicating that G4, I5, and F6 may be involved in VIP1R activation (Supplementary Table 4). These interactions also highly conserved between PACAP38 and cognate residues in PAC1R (Supplementary Table 3).

Compared to the six N-terminal residues of PACAPs, peptide residues from T7 to R14 exhibit different binding modes to VIP1R and PAC1R. Besides identical hydrogen bonds between S11 in PACAPs and D^ECL2^ in two receptors, other polar interactions (D8 and I289^ECL2^, Y13 and D132^1.29^, as well as T136^1.33^ for VIP1R, and S9 and K378^7.35^, S11 and Y211^ECL1^, R12 and D301^ECL2^ for PAC1R) are unique (Fig. 3g, h, Supplementary Table 3). Mutations of I289^ECL2^ to Ala decreased PACAP27 potency in promoting VIP1R to couple with Gs, indicating a specific role of D8 for PACAP27 activity (Supplementary Table 4). Thus, the N-terminus of PACAP27 engages within the helix bundle core in a receptor-specific manner.

The structural studies on VIP1R binding pocket also provide a clue on the potential recognition mechanism of VIP1R by VIP, a peptidic ligand shares highly conserved sequences and bound VIP1R with similar affinities compared to PACAP27^1^. Although the peptide sequences from PACAP27 and VIP are highly conserved (Fig. 3a), these two peptides may interact with VIP1R in a peptide-specific mode. The previous alanine scanning analysis of VIP supported the fact that H1, D3, F6, R12, and R14, identical amino acids at cognate positions of PACAP27, are important for determining the affinity of VIP to VIP1R^30^. Although H1, D3, F6, and R14 are also supposed to be involved in PACAP27-mediated activation of VIP1R, R12 of PACAP27 seems not to form any substantial interaction with residues in VIP1R binding pocket, indicating a distinct VIP1R binding mode for these two peptides.

The structural-based mutagenesis analysis also provides a potential explanation of VIP selectivity for VIP1R over PAC1R. Structurally, G4 in PACAP closely contacts W^5.36b^ in VIP1R. When mutating G4 of PACAP to Ala, the cognate amino acid of VIP, a more significant steric constraint, is generated between the newly mutated A4 and W306^5.36b^ of PAC1R compared to W296^5.36b^ in VIP1R, which may restrict the binding of VIP to PAC1R and lead to a lower selectivity for VIP for PAC1R than VIP1R (Supplementary Fig. 7). This structure feature is coincident with the fact that when replacing A4-V5 dipeptide of VIP by G4-I5 in PACAP, the new VIP analog obtains the ability to bind and activate PAC1R. Similarly, PACAP27 abolished its propensity to bind PAC1R when its G4-I5 sequence was substituted for A4-V5 in VIP^31^.

Together, these observations provide a rationale for understanding VIP1R recognition by PACAP27 and VIP1R-targeted ligand discovery.

### Activation of VIP1R by PACAP27

**Fig. 4:**
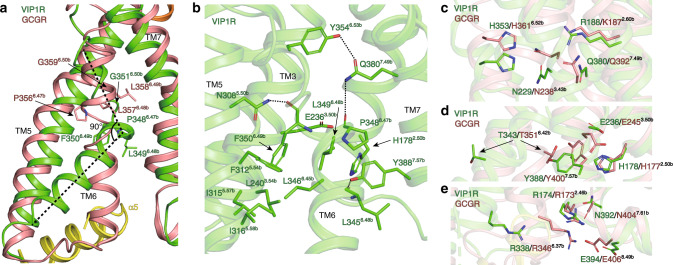
Structure comparisons of active VIP1R with inactive GCGR. **a** The structural alignment of activated VIP1R with inactive GCGR showing the outward bending of the intracellular portion of TM6 of activated VIP1R, which results in a kink at the PxxG motif in TM6 and a ~90° angle between two portions of TM6 of the activated receptor. The TM6 kink in the active VIP1R structure is indicated by a dotted black line. The residues in the conserved PxxG motif in TM6 are shown in stick representation. **b** Polar and hydrophobic interactions that stabilize the kink at TM6 of activated VIP1R. The polar contacts are marked as black dotted lines. The positions of conserved polar residue networks located within VIP1R (green) and inactive GCGR (PDB code:4L6R, colored in salmon): central polar network (**c**), HETY network (**d**), and TM2–6–7–helix 8 network (**e**). Side chains of the residues are shown in stick representation.

The structural hallmark of class B GPCR activation is the much more pronounced outward shift of TM6 than that in class A GPCRs, which is accompanied by the formation of a sharp kink in the middle of the TM6 induced and stabilized by ligand binding. The N-terminal residues H1 and S2 from PACAP27 pack directly against the C-terminus of TM6, and disrupt the helical conformation of the conserved PxxG motif (P348^6.47b^−L349^6.48b^−F350^6.49b^−G351^6.50b^), and create a ~90° sharp kink at the middle of TM6 (Fig. 4a). The kink conformation of TM6 is stabilized by polar interactions between P348^6.47b^ and F350^6.49b^ with the side chains of Q380^7.49b^ and N308^5.50b^, respectively, (Fig. 4b). It is notable that Q380^7.49b^ also forms polar interaction with Y354^6.53b^, a residue at the C-terminal end of the kink, suggesting its critical role in stabilizing the kink conformation of TM6 and the active state of the receptor (Fig. 4b, Supplementary Fig. 8). In addition, compared with conformations of L357^6.48b^ and L358^6.49b^ in the inactive GCGR structure, large conformational rotations of L349^6.48b^ and F350^6.49b^ were induced by the kink of TM6, creating extensive hydrophobic contacts with conserved residues in TM2 (H178^2.50b^), TM3 (L240^3.54b^), TM5 (F312^5.54b^, I315^5.57b^, and I316^5.58b^), TM6 (L346^6.45b^), and TM7 (Y388^7.57b^) to stabilize the kinked TM6 conformation (Fig. 4b).

Compared with the conformation of the inactive GCGR structure^31^, the kink of TM6 and subsequent outward shift of its cytoplasmic end caused a rearrangement of three conserved polar interaction networks, including the central polar network (R188^2.60b^, N229^3.43b^, H353^6.52b^, and Q380^7.49b^), HETY (H178^2.50b^, E236^3.50b^, T343^6.42b^, and Y388^7.57b^), and TM2–6–7–helix 8 (R174^2.46b^, R338^6.37b^, N392^7.61b^, and E394^8.49b^) polar networks (Fig. 4c–e). The residues from the central polar network are involved in peptide ligand binding by the receptor, suggesting that their conformational changes are required for the receptor to facilitate the peptide ligand binding and signal transduction (Fig. 4c and Supplementary Fig. 9). Previous experiments showed that point mutations of VIP1R residues R188^2.60b^, N229^3.43b^, and Q380^7.49b^ severely affect the binding of VIP and VIP-mediated cAMP production^32^, in agreement with our structural data. Interestingly, in many published class B GPCR active structures, these polar network residues are not in close contact with peptide ligands, except for VIP1R, PAC1R, and PTH1R. We observed that residue R188^2.60b^ of VIP1R forms a charge interaction with D3 of PACAP27. The corresponding residue in PAC1R, R199^2.60b^, forms direct polar interactions with N-terminal S2 and D3 of PACAP38. A similar interaction can also be observed between R233^2.60b^ of PTH1R and E4 of LA-PTH (Supplementary Fig. 9). These polar interactions between the peptide and the receptor serve as the structural basis of ligand-induced receptor activation.

Our PACAP27-bound VIP1R–Gs complex structure also exhibits broken HETY and TM2–6–7–helix 8 polar networks, which are caused by the outward movement of the intracellular segment of TM6 that takes away TM6 residues T343^6.42b^ and R338^6.37b^, respectively, from these two networks (Fig. 4d, e). VIP1R contains the conserved HETY motif, which is known to mediate inter-helix interactions of TM2–6–7–helix 8 polar networks in GCGR, PTH1R, and CRFR1. Disruption of this inter-helix interactions has resulted in constitutively active class B GPCRs receptors^33–35^. We, therefore, speculate that TM2–3–6–7 polar networks may also be required for maintaining an inactive conformation, and the breakage of these polar networks may represent the active conformations of VIP1R. Indeed, mutations that disrupt this polar network in VIP1R have resulted in the constitutively active receptor^35–37^.

Taken together, despite the different sequence and physicochemical environment of VIP1R in ligand binding pocket, VIP1R shares a common activation mechanism with other class B GPCRs, which is characterized by a set of conserved residues involved in ligand-induced conformational changes in the receptor helix bundle as well as residues involved in G protein coupling. The polar networks in the helix bundle core, the central polar network, HETY, and TM2–6–7–helix 8 networks, required in maintaining the inactive conformation of the receptor, undergo ligand-induced conformational changes that rearrange the network residues to facilitate the ligand binding and to stabilize the active conformation of the receptor.

### Gs heterotrimer coupling by VIP1R

The overall assembly of the receptor with Gs is remarkably similar to many other class B GPCRs solved to date, with several unique features of receptor-specific interactions with the Gs heterotrimer^21–23,25–28^. The outward moved cytoplasmic end of TM6 and concomitantly shifted TM5 form a cytoplasmic cavity together with TM2, 3, and 7 to accommodate the α5 helix of Gαs. This interface serves as a crucial contact between the receptor and Gs heterotrimer. Additional contacts are observed between extended helix 8 of the receptor and the Gβ subunit of the Gs heterotrimer. ICL3, although invisible in our complex structure, also makes important contributions because residues I328-S331 in the central part of ICL3 are crucial for efficient binding of VIP1R to Gαs^36,37^. Structural alignment of our PACAP27–VIP1R–Gs complex with other class B GPCR–Gs protein complex structures solved to date by superimposing their receptor TM domains reveals different orientations of the Gs heterotrimers with rigid body rotations around the axis of the Gβ subunit (Supplementary Fig. 10a). The structural similarities in the Gs heterotrimer may be influenced by the use of Nb35, which has been used in all the structures of Gs-coupled receptor complexes reported thus far.

**Fig. 5:**
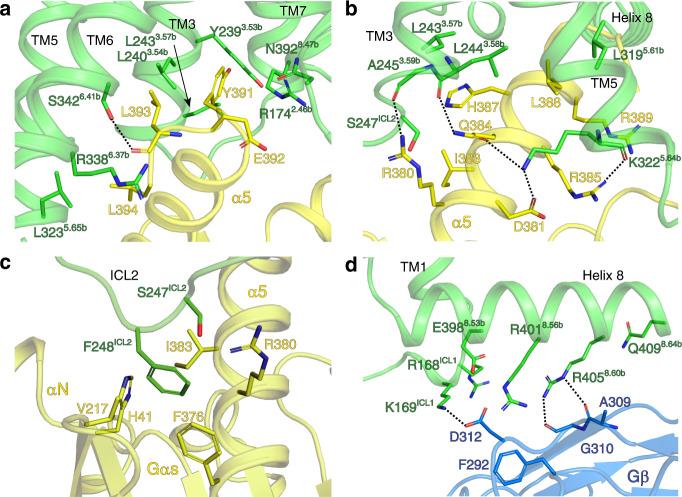
The interactions between VIP1R and Gs heterotrimer. **a**, **b** The binding interface between the cavity on the intracellular side of VIP1R TMD (green) and α5 helix of the Gαs Ras-like domain (yellow). **c** The interface between ICL2 of VIP1R (green) and α5 and αN of the Gαs Ras-like domain (yellow). **d** The interface between helix 8 of VIP1R (green) and Gβ subunit (blue). Residues in VIP1R–Gs interfaces are shown in stick representation.

The VIP1R residues at the interface of the cytoplasmic cavity and the α5 helix of Gαs are highly conserved among class B GPCRs (Supplementary Fig. 8). The polar interactions mediated by these conserved residues on TM3, TM5, and TM6 can also be observed in the interface between VIP1R and Gαs, including an extensive hydrogen bond network formed between α5 helix and the cytoplasmic receptor cavity (Fig. 5a, b). The interface of ICL2 with α5 and αN-β1 junction of Gαs is primarily stabilized by hydrophobic contacts (Fig. 5c). Additional electrostatic contacts presented between K169^ICL1^ and D312 of helix 8 together with hydrogen bonds between R405^8.60b^ and the backbone oxygen of A309 and G310 may further stabilize the interface between helix 8 and Gβ (Fig. 5d).

Compared with other class B GPCR–Gs complex structures, the PACAP27–VIP1R–Gs complex shows different intermolecular interactions at the interface constituted of TM2 and TM3, and the TM7–helix 8 turn of the receptor and α5 of Gαs. Similar to the PTH1R–Gs complex, the VIP1R–Gs complex lacks several polar interactions that are present in other GPCR–Gs complexes between Q390/E392 on α5 and R^2.46^/N^8.57^ as well as residues at 8.48 and 8.49 in the receptors (Supplementary Fig. 10b). The slightly outward movement of the turn between TM7 and helix 8 and the shift of α5 away from the TM7–helix 8 turn lead to a smaller Gαs-buried surface area of VIP1R than those of other class B GPCR–Gs complexes (Supplementary Fig. 10b and Supplementary Table 2). This is consistent with the fact that the VIP1R–Gs complex is not sufficiently stable for cryo-EM studies without NanoBiT tethering.

## Discussion

Here, we report a near-atomic resolution structure of PACAP27-bound VIP1R in complex with Gs, determined by cryo-EM. For successful structure determination, we stabilized the assembly between PACAP27-bound VIP1R and Gs heterotrimer using a developed NanoBiT tethering method. The structure has provided a rationale to understand how PACAP27 interacts with the transmembrane bundle of VIP1R and provides the basis of ligand binding specificity. Structural comparison with other class B GPCRs shed light on the basis of PACAP27 binding as well as a common mechanism of ligand-induced receptor activation and coupling to downstream Gs heterotrimer. As VIP receptors have been identified as potential therapeutic targets for metabolic, inflammatory, and neuronal diseases^38^, this structure presents key information for the rational design of peptides or small molecule compounds to target VIP receptors. In addition, we expect that NanoBiT tethering method can be used to stabilize not only GPCR–G protein complexes but also other unstable macromolecular complexes for structural determination.

## Methods

### Constructs

Human VIP1R (residues 31–437) was cloned into pFastbac with an N-terminal FLAG tag followed by a His8 tag, as well as LgBiT at the C-terminus using homologous recombination (CloneExpress One Step Cloning Kit, Vazyme). The primers used in this study are shown in Supplementary Table 5. The native signal peptide was replaced with the prolactin precursor sequence to increase the protein expression. A dominant-negative bovine Gαs (DNGαs) construct was generated by site-directed mutagenesis to incorporate mutations S54N, G226A, E268A, N271K, K274D, R280K, T284D, and I285T to decrease the affinity of nucleotide-binding and increase the stability of Gαβγ complex^28^. Rat Gβ1 was cloned with an N-terminal His6 tag and a C-terminal SmBiT connected with a 15 residues linker. All three G protein components together with bovine Gγ2 were cloned into a pFastBac vector, respectively.

### Insect cells expression

VIP1R(31–437)–LgBiT fusion, DNGαs, Gβ1–SmBiT fusion, and Gγ2 were coexpressed in *Sf9* insect cells (Invitrogen) using the Bac-to-Bac baculovirus expression system (Thermo Fisher). Cell cultures were grown in ESF 921 serum-free medium (Expression Systems) to a density of 3 × 10^6^ cells mL^−1^ and then infected with baculovirus expressing VIP1R(31–437)–LgBiT fusion, DNGαs, Gβ1–SmBiT fusion, and Gγ2, respectively, at the ratio of 1:1:1:1. The cells were collected by centrifugation at 1000 × *g* (Thermo Fisher, H12000) for 20 min after infection for 48 h, and kept frozen at −80 °C until use.

### Expression and purification of Nb35

Nanobody-35 (Nb35) with a C-terminal His6 tag, was expressed in the periplasm of *E. coli* strain BL21^19^. Cultures of 2 L cells were grown to OD600 = 1.0 at 37 °C in TB media containing 0.1% glucose, 2 mM MgCl_2_, and 100 μg mL^−1^ ampicillin. Then, 1 mM IPTG was added to the medium to induce protein expression for another 4.5 h at 37 °C. Cells were harvested by centrifugation and lysed in ice-cold buffer (50 mM Tris pH 8.0, 12.5 mM EDTA, and 0.125 M sucrose), then centrifuged to remove cell debris. Nb35 was purified by nickel affinity chromatography, followed by size-exclusion chromatography using a HiLoad 16/600 Superdex 75 column, and finally spin concentrated to ~2.5 mg mL^−1^.

### PACAP27–VIP1R–Gs complex formation and purification

Cell pellets from 2 L culture were thawed and lysed in 20 mM HEPES, pH 7.4, 100 mM NaCl, 10% glycerol, 0.25 mM TCEP, 5 mM MgCl_2_, and 5 mM CaCl_2_ supplemented with EDTA-Free Protease Inhibitor Cocktail (Selleck). The VIP1R–Gs complex was formed in membranes by the addition of 10 μM PACAP27 (Synpeptide), 10 μg mL^−1^ Nb35, and 25 mU mL^−1^ apyrase and incubation for 1.5 h at room temperature. Cell membranes were collected by ultracentrifugation at 64,000 × *g* for 35 min. The membranes were then resuspended and solubilized in buffer containing 20 mM HEPES, pH 7.4, 100 mM NaCl, 10% glycerol, 0.25 mM TCEP, 5 mM MgCl_2_, 5 mM CaCl_2_, and 0.5% (w/v) lauryl maltose neopentylglycol (LMNG, Anatrace), 0.1% (w/v) cholesteryl hemisuccinate TRIS salt (CHS, Anatrace), 5 µM PACAP27, and 25 mU mL^−1^ apyrase for 3 h at 4 °C. The supernatant was collected by centrifugation at 80,000 × *g* for 40 min and then incubated with 3 mL pre-equilibrated Nickel-NTA resin for 2 h at 4 °C. After batch binding, the resin was loaded into a plastic gravity flow column and washed with ten column volumes of 20 mM HEPES, pH 7.4, 100 mM NaCl, 40 mM imidazole, 10% glycerol, 0.25 mM TCEP, 2 mM MgCl_2_, 2 mM CaCl_2_, 0.01% (w/v) LMNG, 0.01% glyco-diosgenin (GDN, Anatrace) and 0.002% (w/v) CHS, 5 μM PACAP27 and eluted with five column volumes of the same buffer plus 250 mM imidazole. The Ni-NTA-purified fraction was immobilized by batch binding to M1 anti-Flag affinity resin overnight at 4 °C. Next day, the M1 anti-Flag affinity resin was washed with five column volumes of 20 mM HEPES, pH 7.4, 100 mM NaCl, 10% glycerol, 0.25 mM TCEP, 2 mM MgCl_2_, 2 mM CaCl_2_, 0.01% (w/v) LMNG, 0.01% GDN (Anatrace) and 0.002% (w/v) CHS, 5 μM PACAP27 and eluted with five column volumes of the same buffer plus 0.2 mg mL^−1^ Flag peptide. The complex was then concentrated using an Amicon Ultra Centrifugal Filter (MWCO 100 kDa) and injected onto a Superdex 200 10/300 GL column (GE Healthcare) equilibrated in the buffer containing 20 mM HEPES, pH 7.4, 100 mM NaCl, 2 mM MgCl_2_, 2 mM CaCl_2_, 0.0015% (w/v) LMNG, 0.0005% GDN, 0.0003% (w/v) CHS, 5 μM PACAP27, and 100 μM TCEP. The complex fractions were collected and concentrated for electron microscopy experiments. The final yield of the purified complex was ~0.2 mg per liter of insect cell culture.

### CCR7–Gi–scfv16 complex expression and purification

The cDNA of human WT CCR7 was cloned into pFastbac with an LgBiT inserted at the C-terminal of CCR7. The CCR7–LgBiT was followed by a C-terminal double MBP and His8 tag to facilitate purification. Receptor, human DNGαi (G203A, A326S), rat Gβ1, bovine Gγ2, and scfv16 were coexpressed and assembled in *Sf9* insect cells. The CCR7–Gi–scfv16 complex was purified substantially in the same way described above except for the MBP instead of M1 anti-Flag affinity purification.

### Negative-stain electron microscopy screening

For preparing 0.75% uranyl formate solution, weigh out 37.5 mg of uranyl formate into a small beaker, add 5 mL of boiling water and stir for 5 min in the dark, add 10 μL of 5 M NaOH, continue stirring for 5 min, and finally filter the solution using a syringe filter^39^. 300-mesh copper grids with carbon film (Electron Microscopy Sciences) were glow-discharged (PELCO easiGlow™ Glow Discharge Cleaning System) for 1 min at 25 mA before 3.5 µL purified complex was applied to the grids and incubated for 30 s. After blotting the sample using filter paper, the grid surface was touched on two drops of 40 µL 0.75% uranyl formate, and then the grids were stained on the third drop of uranyl formate with gentle stirring for 40 s. Stained grids were blotted to remove excess stain. Negative-stain data collection was carried out on a Tecnai G2 Spirit transmission electron microscopy (Thermo FEI) operating at 120 kV. Images were collected at a nominal magnification of 105,000 (3.1 Å pixel size) within a −0.5 to −2.5 µm defocus range.

### Cryo-EM data acquisition

The purified PACAP27–VIP1R–Gs complex (3.0 μL) at a concentration of 4–5 mg mL^−1^ was applied to glow-discharged holey carbon grids (Quantifoil R1.2/1.3, 200 mesh), and subsequently vitrified using a Vitrobot Mark IV (Thermo Fisher Scientific). Cryo-EM images were collected on a Titan Krios equipped with a Gatan K2 Summit direct electron detector. The microscope was operated at 300 kV accelerating voltage, at a nominal magnification of ×29,000 in counting mode, corresponding to a pixel size of 1.014 Å. In total, 4215 image stacks were obtained at the dose rate of about eight electrons per Å^2^ per second with a defocus range of −1.5 to −2.3 μm. The total exposure time was set to 8 s with intermediate frames recorded every 0.2 s, resulting in an accumulated dose of 64 electrons per Å^2^.

### Image processing and 3D reconstruction

Dose-fractionated image stacks were subjected to beam-induced motion correction and dose-weighting using MotionCor2.1^40^. A sum of all frames, filtered according to the exposure dose, in each image stack was used for further processing. Contrast transfer function parameters for each micrograph were determined by Gctf v1.06^41^. The further data processing was performed in RELION-3.0-beta2^42^. Particle selection, two-dimensional classification and the first round of three-dimensional classification were performed on a binned dataset with a pixel size of 2.028 Å. Auto-picking yielded 2,547,930 particle projections that were sequentially subjected to reference-free two-dimensional classification and produced 2,460,220 projections for further processing. This step barely discard false-positive particles or particles categorized in poorly defined classes, indicating the complex stability of the sample generated using NanoBiT tethering method developed in this study. This subset of particle projections was subjected to consecutive rounds of 3D classifications with a pixel size of 2.028 Å. A selected subset containing 131,263 projections was used to obtain the final map using a pixel size of 1.014 Å. After the last round of refinement, the final map has an indicated global resolution of 3.2 Å at a Fourier shell correlation of 0.143. Local resolution was determined using the Bsoft package with half maps as input maps^43^.

### Model building and refinement

The cryo-EM structure of PTH1R–Gs–Nb35 complex (PDB code 6NBF) was used as the start for model rebuilding and refinement against the electron microscopy map. The model was docked into the electron microscopy density map using Chimera^44^, followed by iterative manual adjustment and rebuilding in COOT^45^. Real space refinement was performed using phenix.real_space_refine from Phenix program package^46^. The model statistics were validated using MolProbity^47^. Structural figures were prepared in Chimera and PyMOL (https://pymol.org/2/). The final refinement statistics are provided in Supplementary Table 1.

### cAMP accumulation assay

The full-length VIP1R(31–457) and VIP1R mutants was cloned into pcDNA6.0 vector (Invitrogen) with a FLAG tag at its N-terminus (see Supplementary Table 5 for a list of primers used in this study). CHO-K1 cells (ATCC, #CCL-61) were cultured in Ham’s F-12 Nutrient Mix (Gibco) supplemented with 10% (w/v) fetal bovine serum. Cells were maintained at 37 °C in a 5% CO_2_ incubator with 100,000 cells per well in a 12-well plate. Cells were grown overnight and then transfected with 1 μg VIP1R constructs by FuGENE^®^ HD transfection reagent (DNA/FuGENE^®^ HD ratio of 1:3) in each well. After 24 h, the transfected cells were seeded onto 384-well microtiter plates (3000 cells per well). cAMP accumulation was measured using the LANCE cAMP kit (PerkinElmer) according to the manufacturer’s instructions with different concentrations of peptides. Fluorescence signals were then measured at 620 and 665 nm by an Envision multilabel plate reader (PerkinElmer). Data presented are means ± SEM of at least three independent experiments.

### Detection of surface expression of VIP1R mutants

The VIP1R mutants were cloned into pcDNA6.0 vector (Invitrogen) with a FLAG tag at its N-terminus. The cell seeding and transfection follow the same method as cAMP accumulation assay. After 24 h of transfection, cells were washed once with PBS and digested with 0.2% (w/v) EDTA in PBS. Cells were blocked with PBS containing 5% (w/v) BSA for 15 min at room temperature and then incubated with primary anti-Flag antibody (diluted with PBS containing 5% BSA at a ratio of 1:300, Sigma) for 1 h at room temperature. Thereafter, cells were washed three times with PBS containing 1% (w/v) BSA before incubating with anti-mouse Alexa-488-conjugated secondary antibody (diluted with PBS containing 5% BSA at a ratio of 1:1000, Invitrogen) at 4 °C in the dark for 1 h. After another three times wash, cells were resuspended, and fluorescence intensity was quantified in a BD Accuri C6 flow cytometer system (BD Biosciences) at excitation 488 nm and emission 519 nm. Approximately 10,000 cellular events per sample were collected and data were normalized to WT.

### Dynamic light scattering

DLS sample was prepared at about 0.2–1.0 mg mL^−1^ and equilibrated for 5 min before loading 10 μL onto the DynaPro NanoStar (Wyatt Technology). For thermostability assay, the intensity was read with a thermal ramp from 25 to 75 °C with a ramp rate of 2 °C min^−1^. All data acquisition and analysis were performed by the Dynamics software.

### Reporting summary

Further information on research design is available in the Nature Research Reporting Summary linked to this article.

## Supplementary information

Supplementary Information

Peer Review File

Reporting Summary

## Data Availability

Data supporting the findings of this manuscript are available from the corresponding authors upon reasonable request. A reporting summary for this article is available as a Supplementary Information file. Source data are provided with this paper. Density map and structure coordinate have been deposited to the Electron Microscopy Database and the Protein Data Bank with the accession number of EMD-21249, PDB6VN7 for the PACAP27–VIP1R–Gs complex.

## Source data

Source Data
