# Supplementary material for: Cryo-EM structure of an activated VIP1 receptor-G protein complex revealed by a NanoBiT tethering strategy
Source: Nat Commun. 2020 Aug 17;11:4121. doi: 10.1038/s41467-020-17933-8 (PMC7431577; doi:10.1038/s41467-020-17933-8)
Supplement: Supplementary file 1 — Supplementary Information [file 41467_2020_17933_MOESM1_ESM.pdf]

Supplementary Information

Cryo-EM structure of an activated VIP1 receptor-G protein complex revealed by a NanoBiT tethering strategy

Duan et al.

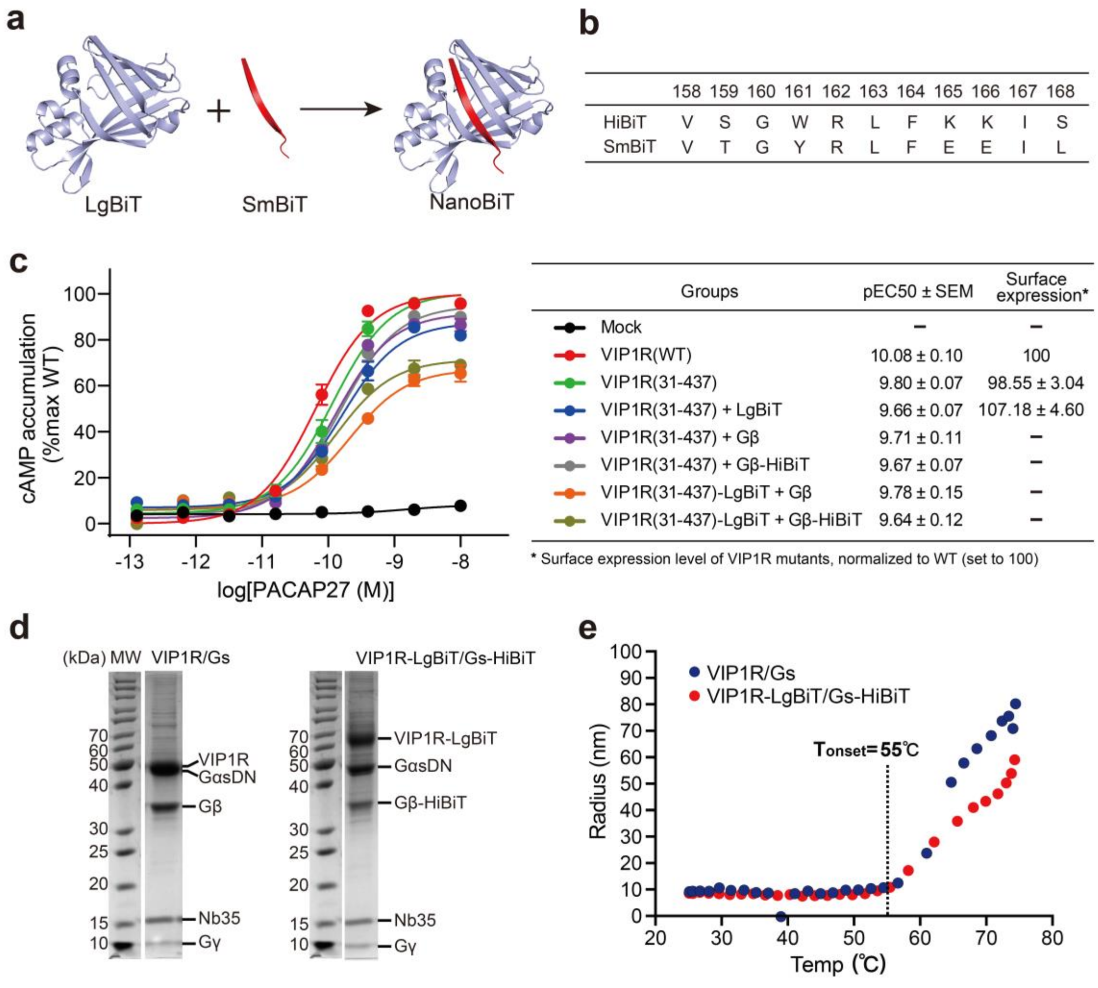

Supplementary Figure 1. Characterization of VIP1R and its Gs complex

a, Schematic diagram of the NanoBiT system. **b**, Sequence comparison of HiBiT and SmBiT. **c**, PACAP27 concentration-cAMP response curves in cells expressing wild-type VIP1R, VIP1R(31-437), and combinations of LgBiT-fused VIP1R(31-437) and HiBiT-fused Gβ subunit. pEC50s are listed in the table. The surface expression of VIP1R mutants were assessed by flow cytometry. The data were normalized to WT, which was set to 100. **d**, SDS-PAGE analysis of truncated VIP1R (31-437)-Gs and VIP1R (31-437)-LgBiT-Gs-HiBiT complexes. **e**, Thermostability analysis of VIP1R (31-437)-Gs and VIP1R (31-437)-LgBiT-Gs-HiBiT complexes by dynamic light scattering (DLS). The hydrodynamic radii of the corresponding VIP1R complexes were monitored as a function of temperature. Source data are provided as a Source Data file.

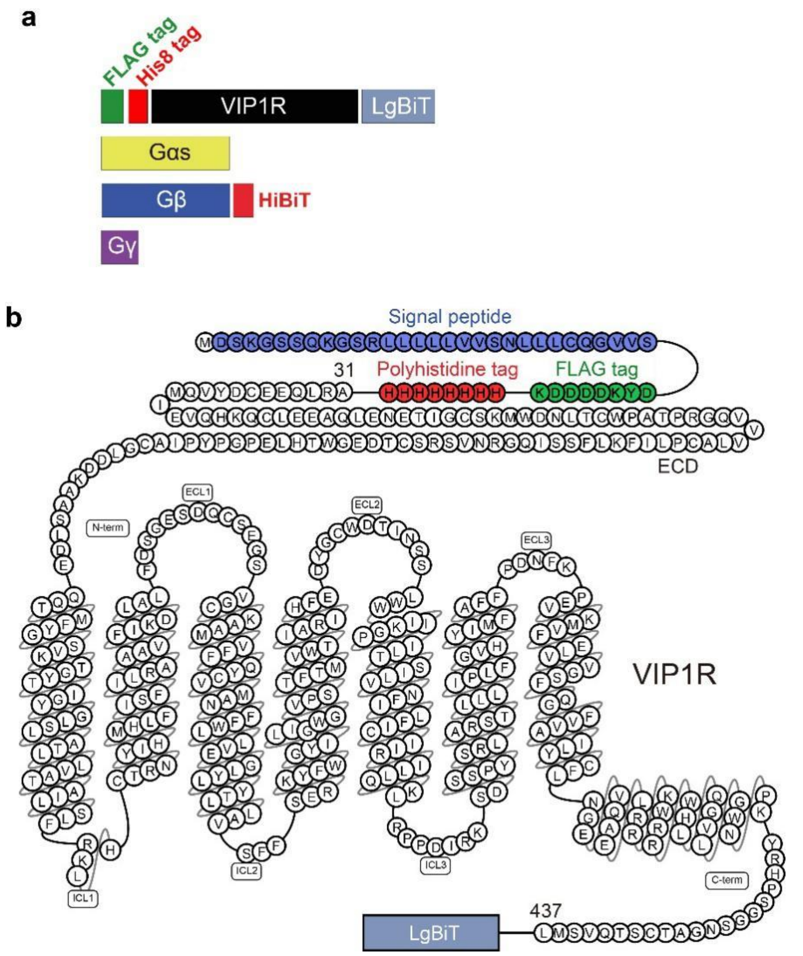

Supplementary Figure 2. Schematic diagrams of human VIP1R-Gs complex constructs

a, Schematic diagram of human VIP1R-Gs complex constructs used in this study. **b**, Snake-plot diagram of the VIP1R-LgBiT construct.

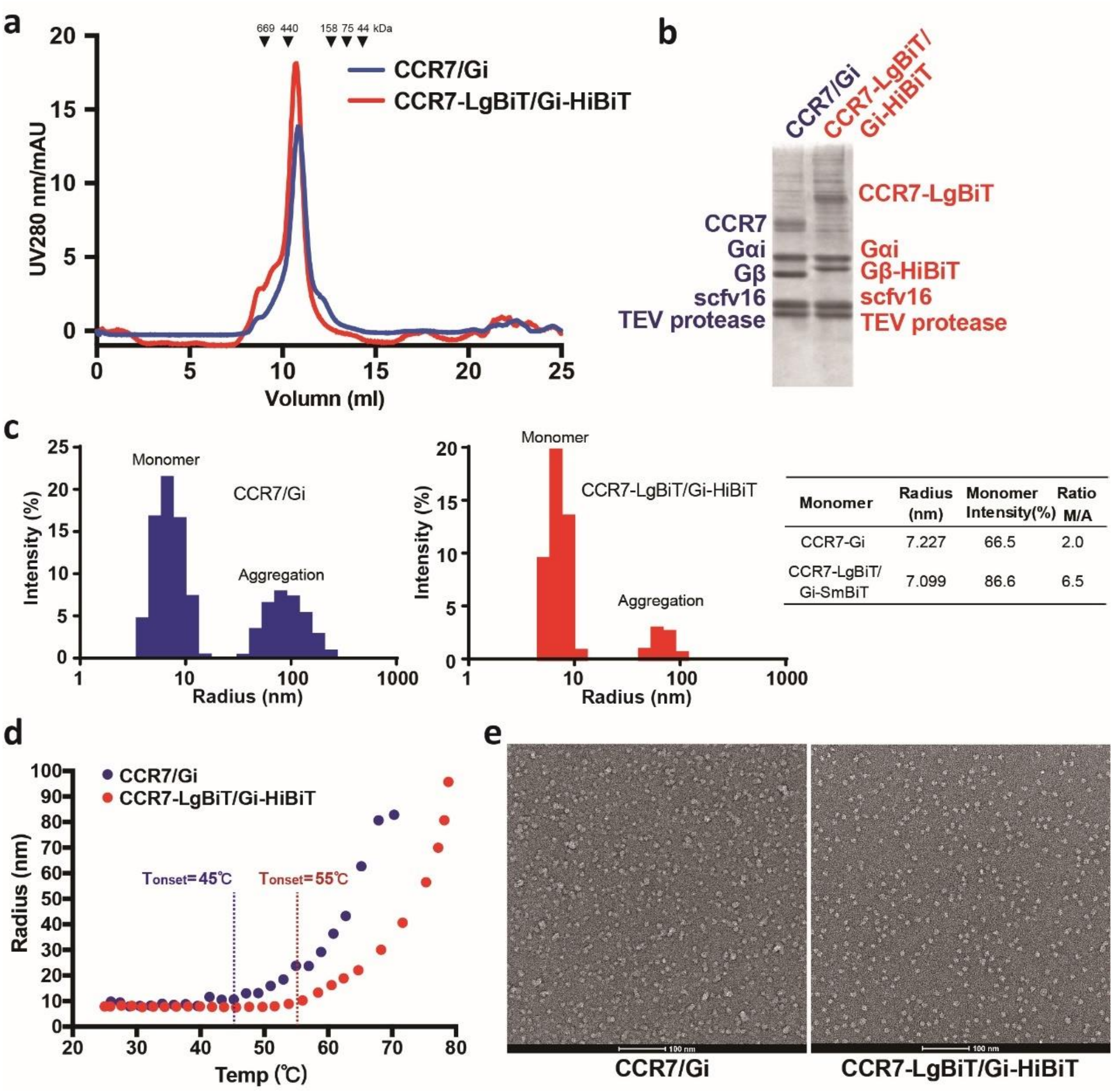

Supplementary Figure 3. Characterization of a CCR7-LgBiT/Gs-HiBiT complex

a, Size-exclusion chromatography elution profiles of the CCR7-Gs and CCR7-LgBiT/Gs-HiBiT complexes. **b**, SDS-PAGE analysis of the eluted complexes. **c**, Dynamic light scattering (DLS) size distribution histogram of CCR7/Gi and CCR7-LgBiT/Gi-HiBiT complexes. Values of Radius, % intensity of monomer, and Ratio of monomer/aggretation (M/A) are listed. **d**, Thermostability analysis of CCR7/Gi and CCR7-LgBiT/Gi-HiBiT complexes by DLS. The hydrodynamic radii of corresponding CCR7-Gi complexes were monitored as a function of temperature. NanoBiT-tethered complex exhibited a significantly enhanced thermostability with its T_{onset} value increased from 45 °C to 55 °C. **e**, Representative negative staining images of the corresponding complexes. The scale bar is 100 nm. Source data are provided as a Source Data file.

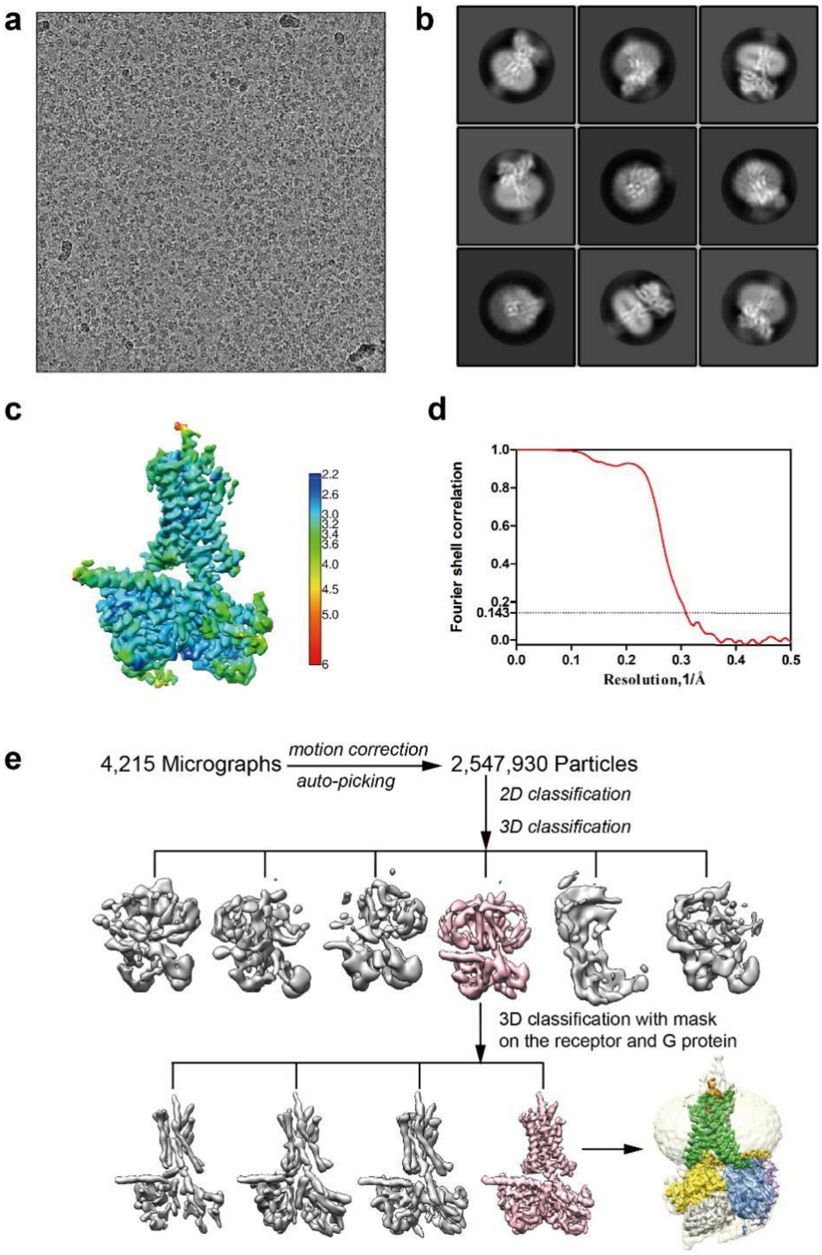

Supplementary Figure 4. Single particle cryo-EM analysis of the PACAP27-VIP1R-Gs complex

a, Representative cryo-EM micrograph of the PACAP27-VIP1R-Gs complex. The scale bar is 30 nm. **b**, Representative 2D class averages displaying distinct secondary structural features from different views. The scale bar is 5 nm. **c**, Cryo-EM map of the PACAP27-VIP1R-Gs complex colored by local resolutions from 2.2 Å (blue) to 6 Å (red). **d**, The "Gold-standard" Fourier shell correlation curve indicates that the overall resolution of the electron density map of the complex is 3.2 Å. **e**, Flowchart of cryo-EM data analysis.

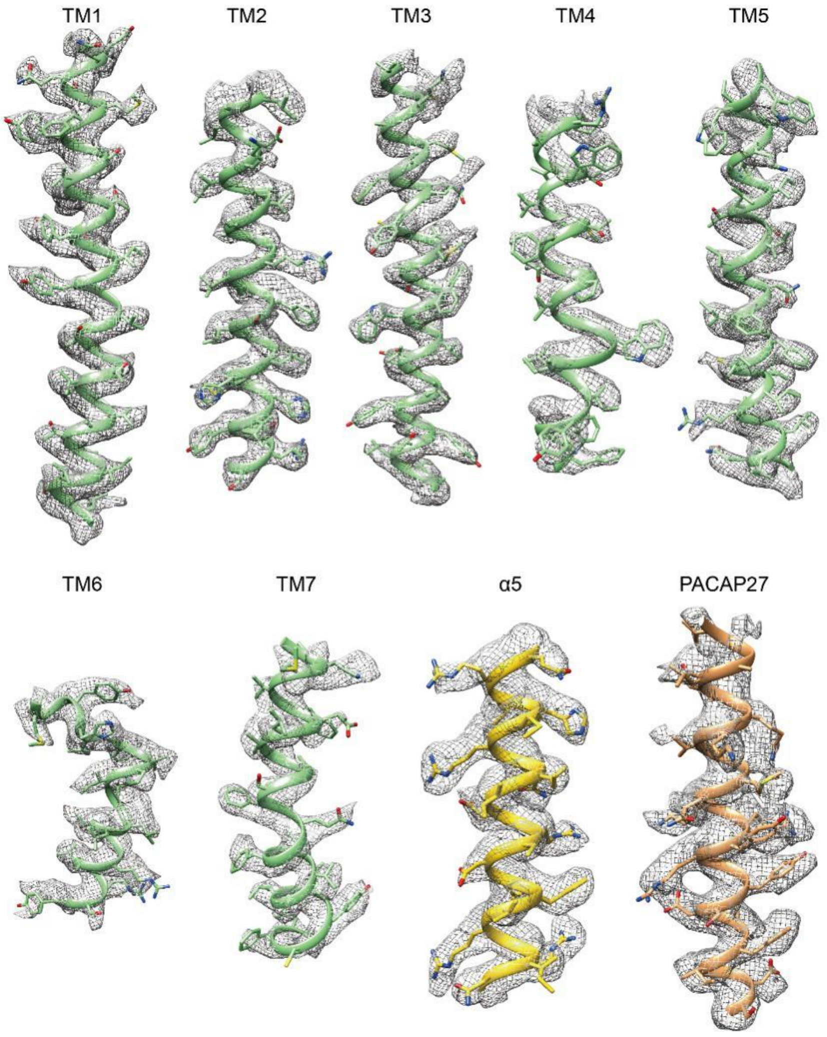

Supplementary Figure 5. Cryo-EM density map and the model of the PACAP27-VIP1R-Gs complex

The regions of the cryo-EM density map with all transmembrane helices, PACAP27 and α5 helix of the Gαs Ras-like domain are shown.

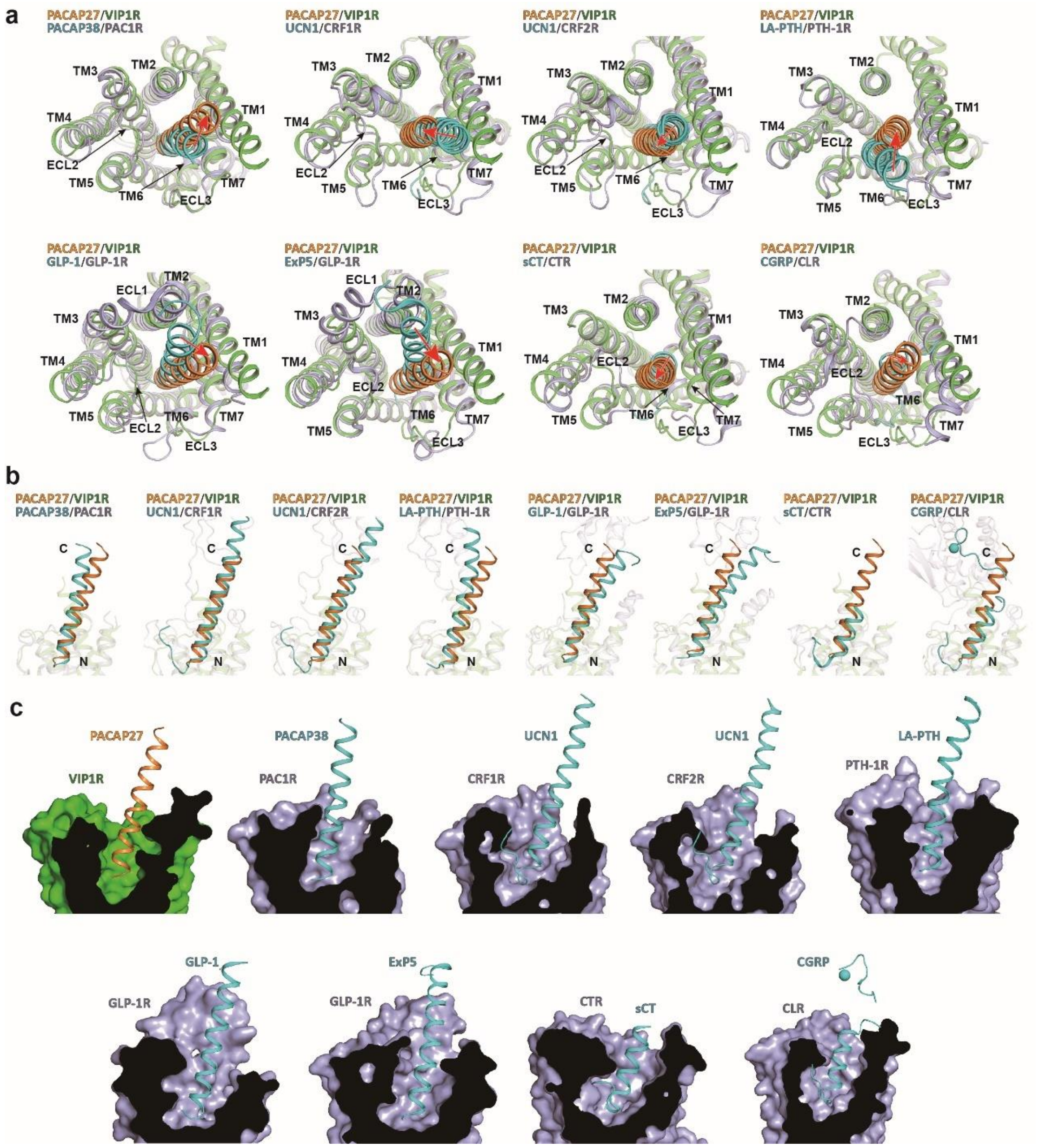

Supplementary Figure 6. Structure comparison of the VIP1R ligand binding pocket with those of other class B GPCRs solved to date

a,**b**, Comparison of PACAP27 (orange) with the peptide ligands (light blue) of other class B GPCRs. **a**, The top views. Red arrows indicate the directions of the shift of N-terminus of PACAP27. **b**, The side views. **c**, The cross-section views of the peptide ligand-binding pockets in the TM bundle of class B GPCRs. The structures are shown in the figure and their PDB codes: PACAP38-PAC1R-Gs, 6P9Y; UCN1-CRF1R-Gs, 6PB0; UCN1-CRF2R-Gs, 6PB1; LA-PTH-PTH1R-Gs, 6NBF; GLP-1-GLP-1R-Gs, 5VAI; ExP5-GLP-1R-Gs, 6B3J; sCT-CTG-Gs, 6NIY; and CGRP-CLR-Gs, 6E3Y.

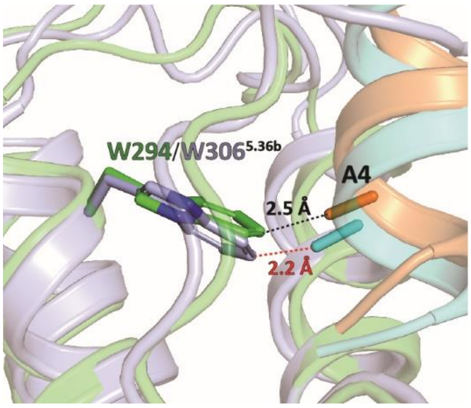

Supplementary Figure 7. A structural model comparison for steric hindrance between G4 of VIP and W$^{5.36b}$ of VIP1R and PAC1R

The mutation from glycine of PACAP at position 4 to alanine, the cognate amino acid in VIP, is generated by the SPDBV (Swiss PDB Viewer) program. The structures of PACAP27-VIP1R and PACAP38-PAC1R complex (PDB code:6P9Y) serve as models for mutations. VIP bound to VIP1R (green) is colored in orange, and VIP bound to PAC1R (lightblue) is shown in cyan. The distances between G4 of VIP and W$^{5.36b}$ of the receptors are labeled in black (VIP1R) and red (PAC1R). Compared to VIP1R, a more significant steric hindrance is indicated between G4 of VIP and W$^{5.36b}$ of PAC1R.

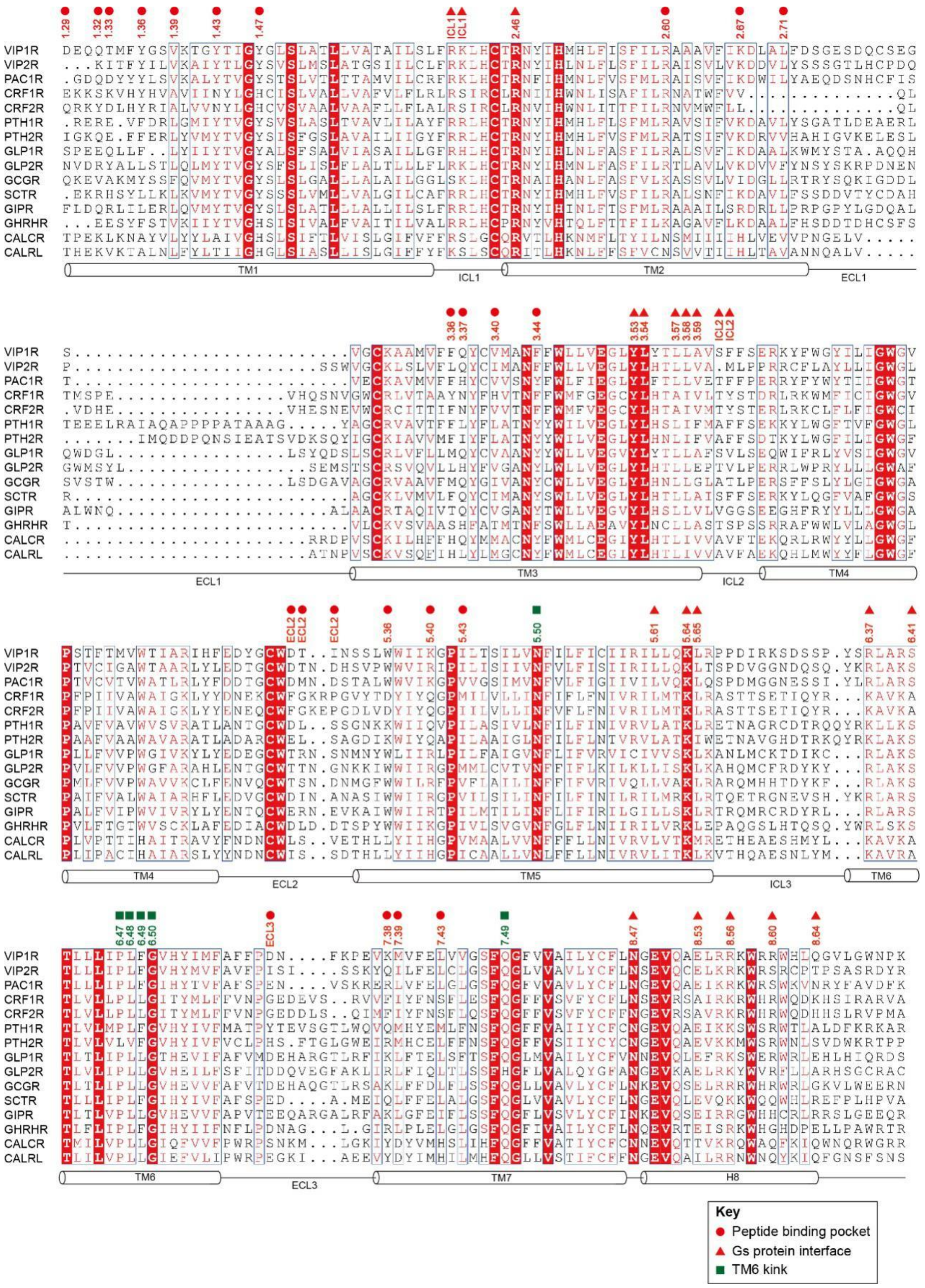

Supplementary Figure 8. Sequence alignment of class B GPCRs excluding the ECD regions

Highlighted are residues consisting of the peptide-binding pockets (red circle), the Gs coupling interface (red triangle), and the TM6 kink of VIP1R (green rectangle). Secondary structure elements are annotated underneath the sequences based on the structure of the PACAP27-VIP1R-Gs complex.

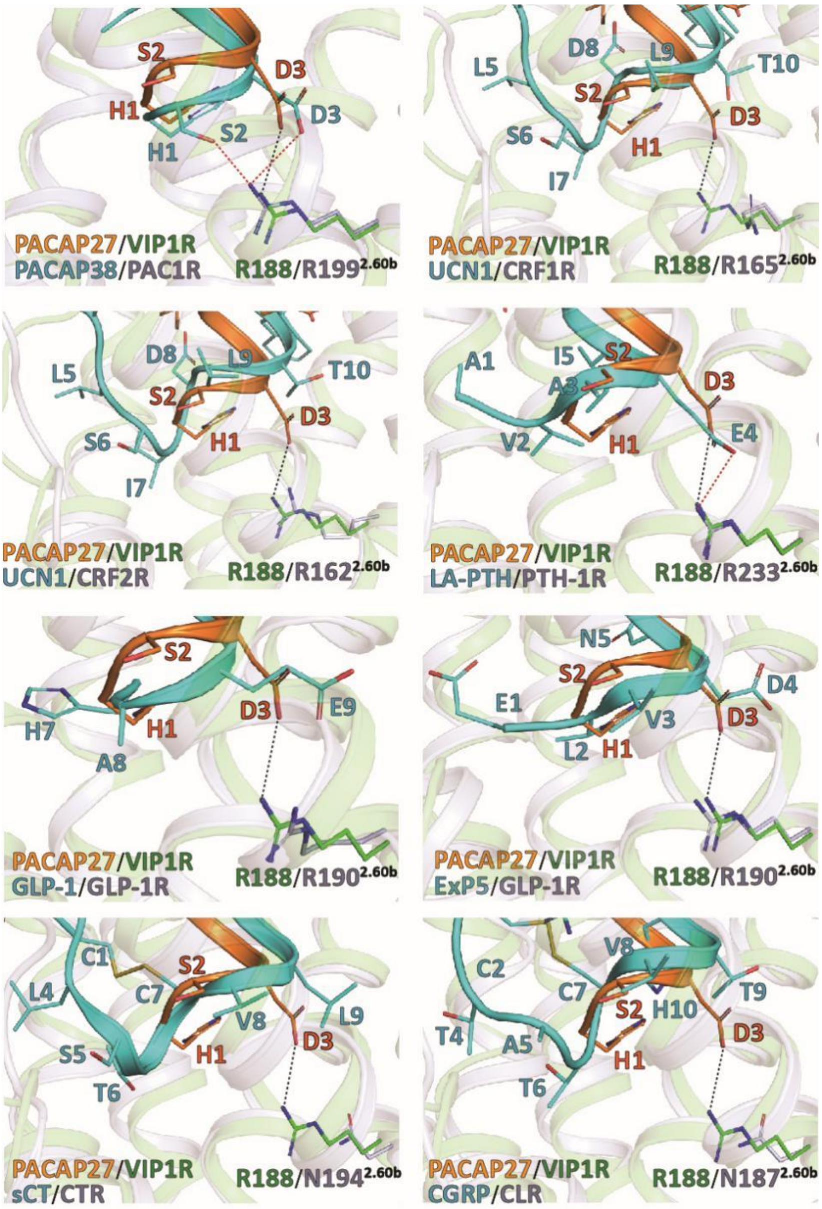

Supplementary Figure 9. The interactions between N-terminal peptide residues and the conserved receptor residues at position 2.60b

Comparison of the interactions between the residues at N-termini of the peptide ligands and R/N$^{2.60b}$ in the central polar networks of class B GPCRs. VIP1R is colored in green; PACAP27, in orange; GPCRs except for VIP1R, in light blue; and peptidic ligands binding to other GPCRs except for VIP1R, in cyan. H-bond formed between R188$^{2.60b}$ of VIP1R and D3 of PACAP27 is labeled as a black dotted line. The corresponding H-bonds formed between R199$^{2.60b}$ (PAC1R), S2 and D3 (PACAP38), as well as R233$^{2.60b}$ (PTH1R) and E4 (LA-PTH) are shown as red dotted lines. The PDB codes of the structures: PACAP38-PAC1R-Gs, 6P9Y; UCN1-CRF1R-Gs, 6PB0; UCN1-CRF2R-Gs, 6PB1; LA-PTH-PTH1R-Gs, 6NBF; GLP-1-GLP-1R-Gs, 5VAI; ExP5-GLP-1R-Gs, 6B3J; sCT-CTG-Gs, 6NIY; and CGRP-CLR-Gs, 6E3Y.

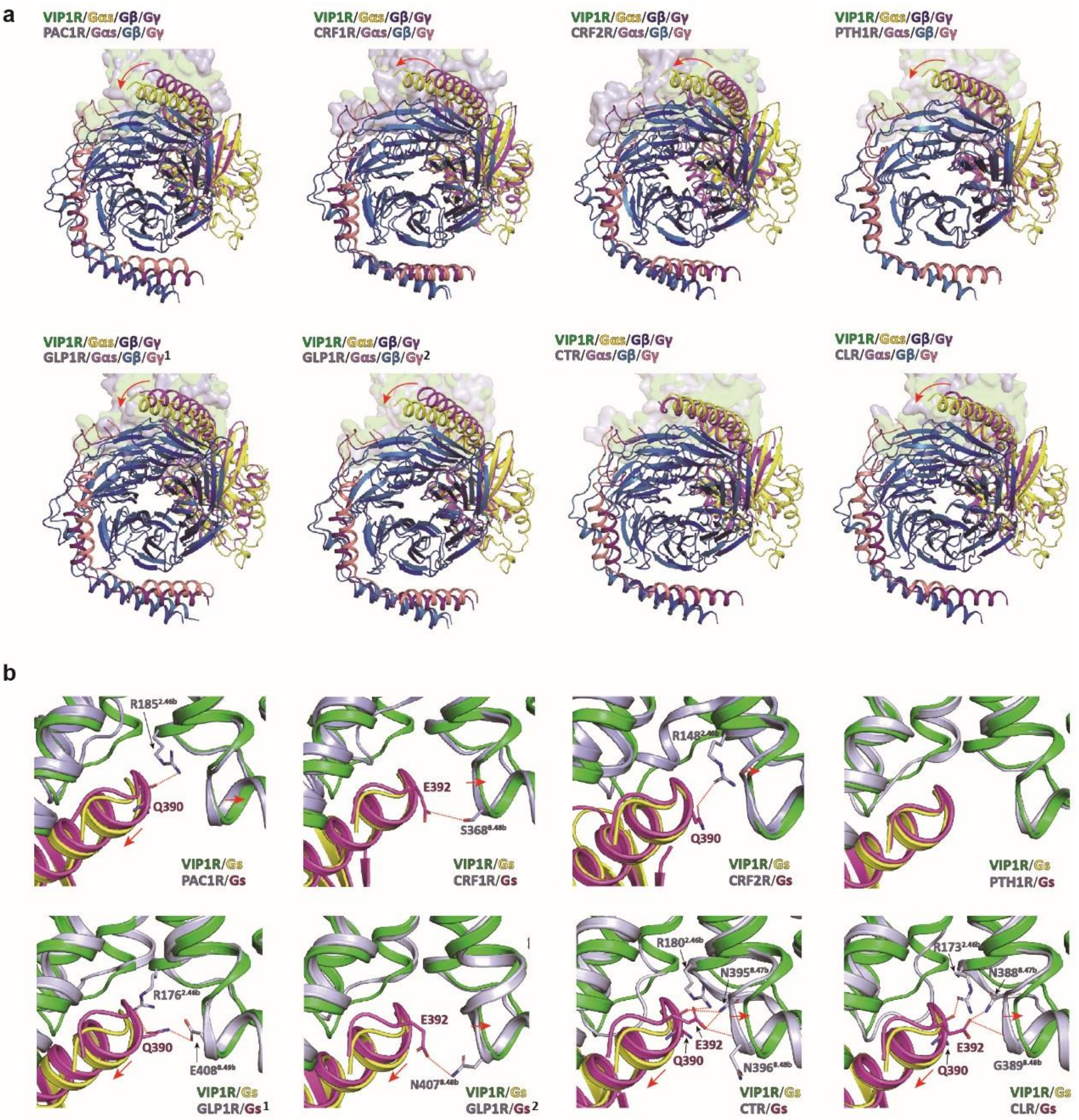

Supplementary Figure 10. Conformational comparisons of VIP1R-Gs complex with other class B GPCR-Gs complexes solved to date

a, Structural alignment of PACAP27-VIP1R-Gs complex with other class B GPCR-Gs protein complexes by superimposing their receptor TM domains reveals different orientations of the Gs protein in its complexes with different class B GPCRs. VIP1R is colored in green, Gαs in yellow, Gβ in blue, Gγ in purple for VIP1R/Gs complex; for other class B GPCR-Gs complexes, the receptor is colored in light blue, Gαs in magenta, Gβ in sky blue, and Gγ in salmon. **b**, Structural comparison of the interfaces between the receptors and α5 helix of Gαs. The polar interactions between TM2, 3, and H7-Helix8 turn of the receptors and α5 helix of Gαs are presented as red dotted lines. The red arrows denote the shift of H7-Hexlix8 turn and α5 helix of the VIP1R-Gs complex compared to other class B GPCR-Gs complexes. Superscript 1 stands for the GLP-1-GLP-1R-Gs complex, and superscript 2 refers to ExP5-GLP-1R-Gs complex. The PDB codes of the structures: PACAP38-PAC1R-Gs, 6P9Y; UCN1-CRF1R-Gs, 6PB0; UCN1-CRF2R-Gs, 6PB1; LA-PTH-PTH1R-Gs, 6NBF; GLP-1-GLP-1R-Gs, 5VAI; ExP5-GLP-1R-Gs, 6B3J; sCT-CTG-Gs, 6NIY; and CGRP-CLR-Gs, 6E3Y.

Supplementary Table 1. Cryo-EM data collection, model refinement, and validation statistics

PACAP27- VPAC1R-Gs-Nb35	Value
**Data collection and processing**	
Magnification	49310
Voltage (kV)	300
Electron exposure (e^{-}/Å^2)	64
Defocus range (μm)	-1.5 ~ -2.3
Pixel size (Å)	1.014
Symmetry imposed	C1
Initial particle projections (no.)	2,547,930
Final particle projections (no.)	131,263
Map resolution (Å)	3.2
FSC threshold	0.143
Map resolution range (Å)	2.5-6
**Refinement**	
Initial model used (PDB code)	6NBH
Model resolution (Å)	3.2
FSC threshold	0.143
Model resolution range (Å)	50-3.2
Map sharpening *B* factor (Å^2)	-95
Model composition	
Non-hydrogen atoms	8683
Protein residues	860
Lipids	10
B factors (Å^2)	
Protein	87.8
Lipids	97.2
R.m.s. deviations	
Bond lengths (Å)	0.005
Bond angles (°)	1.135
Validation	
MolProbity score	1.24
Clashscore	3.85
Poor rotamers (%)	0.11
Ramachandran plot	
Favored (%)	97.7
Allowed (%)	2.3
Disallowed (%)	0

Supplementary Table 2. The volumes of the TMD peptide-binding pockets in class B GPCRs and buried areas of the receptor-G protein interfaces

The volumes of the receptor TMD binding pockets were calculated by Pymol and MOLE. Briefly, all the receptors were firstly prepared by adding hydrogens and optimizing atom positions. PyMOL was then used to select TMD peptide pocket based on the distance between the receptor and peptide. The pocket volumes were finally calculated by MOLE. BSA, the Buried Surface Area, calculated by Chimera. The PDB codes of the calculated structures: PACAP38-PAC1R-Gs, 6P9Y; UCN1-CRF1R-Gs, 6PB0; UCN1-CRF2R-Gs, 6PB1; LA-PTH-PTH1R-Gs, 6NBF; GLP-1-GLP-1R-Gs, 5VAI; ExP5-GLP-1R-Gs, 6B3J; sCT-CTG-Gs, 6NIY; and CGRP-CLR-Gs, 6E3Y.

Receptor complexes	Volume (Å^3)	BSA (Å^2)		
	TMD peptide-binding pocket	Receptor-Gs	Receptor-Gαs	Receptor-Gβ
PACAP27-VIP1R-Gs	3261	1350.18	927.85	309.43
PACAP38-PAC1R-Gs	3246	1345.2	1138.1	207.1
UCN1-CRFR1-Gs	3373	1422.44	1056	366.44
UCN1-CRFR2-Gs	3315	1492.11	1040.7	451.41
LA-PTH-PTH1R-Gs	3576	1203.87	939.96	263.91
GLP1-GLP1R-Gs	3682	1473.02	1212.1	260.92
ExP5-GLP1R-Gs	3657	1156.76	943.68	213.08
sCT-CTR-Gs	3521	1318.48	1055.6	262.88
CGRP-CLR-Gs	3523	1299.69	1078.9	220.79

Supplementary Table 3. Comparison of the interactions of PACAP N-terminus with VIP1R and PAC1R

Superscripts refer to the Wootten conserved class B numbering system. Residues within 4 Å are shown. Sites of conserved residues utilized by two receptors are shadowed in gold. Sites of residues with similar hydrophobic properties are labeled in yellow. Residues in receptors forming hydrogen bonds with PACAP are highlighted in green. The PDB code of PAC1R is 6P9Y.

PACAP	Numbering	VIP1R	PAC1R
H1	3.37	Q223	H234
	3.40	V226	V237
	3.44	F230	Y241
	5.36	W294	W306
	5.40	K298	K310
	5.43	I301	V313
S2	2.60		R199
	3.44		Y241
	7.39		L382
	7.43	L374	L386
D3	1.47		Y161
	2.60	R188	R199
	2.64		V203
	3.36	F222	F233
	7.43	L374	L386
G4	5.36	W294	W306
I5	ECL2	I289	
	5.36	W294	W306
	7.39	M370	L382
F6	1.36	Y139	Y150
	1.39	V142	V153
	1.43	Y146	Y157
	7.39	M370	L382
	7.43	L374	L386
T7	ECL1		Y211
D8	ECL2	I289	
S9	1.36	Y139	Y150
	7.35		K378
	7.39	M370	
Y10	1.36	Y139	Y150
	1.37		L151
	2.71	L199	
	ECL1		Y211
S11	ECL1		Y211
	ECL2	D287	D298
	ECL2		M299
R12	ECL2		M299
	ECL2		D301
Y13	1.29	D132	
	1.32	Q135	
	1.33	T136	
	1.34		Y148
	1.36		Y150
R14	2.71	L199	

Supplementary Table 4. PACAP27-induced activation on wild type and VIP1R with site-directed mutations

The LANCE based cAMP accumulation assay was performed to evaluate the activation of corresponding VIP1R mutants. Data represent mean pEC_{50} (pEC_{50} ± SEM). Experiments were performed in triplicate. *P<0.05, **P<0.01 versus WT. The surface expression of VIP1R mutants were assessed by flow cytometry, and the data were normalized to WT, which was set to 100. Source data are provided as a Source Data file.

Region	Construct	pEC_{50} ± SEM	Surface expression (%WT)
	WT	10.45 ± 0.15	100
TM1	$Q135^{1.32b}A$	10.31 ± 0.07	122.99 ± 11.35
	$T136^{1.33b}A$	10.26 ± 0.09	85.18 ± 10.38
	$Y139^{1.36b}A$	9.51 ± 0.05**	55.24 ± 9.23
	$V142^{1.39b}A$	10.23 ± 0.08	95.09 ± 4.17
	$Y146^{1.43b}A$	9.27 ± 0.12**	82.83 ± 16.38
TM2	$R188^{2.60b}A$	8.15 ± 0.08**	69.53 ± 2.41
	$L199^{2.71b}A$	9.69 ± 0.08*	84.84 ± 20.60
TM3	$F222^{3.36b}A$	9.44 ± 0.19*	71.48 ± 8.99
	$Q223^{3.37b}A$	9.13 ± 0.12**	108.32 ± 15.10
	$V226^{3.40b}A$	10.01 ± 0.10	69.59 ± 10.93
	$F230^{3.44b}A$	10.47 ± 0.19	70.89 ± 15.09
ECL2	$I289^{ECL2}A$	9.90 ± 0.05*	102.97 ± 18.88
TM5	$W294^{5.36b}A$	9.35 ± 0.13*	116.54 ± 3.28
	$I301^{5.43b}A$	10.22 ± 0.04	73.01 ± 10.76
TM7	$M370^{7.39b}A$	10.05 ± 0.16	118.41 ± 6.42
	$L374^{7.43b}A$	10.16 ± 0.14	108.82 ± 12.08

Supplementary Table 5. List of primer sequences used in this study

Oligonucleotide name	Oligonucleotide sequence (5'→3')	Cloning method	Product
VIP1R(31-437) Forward	CACCATCACCATCACGCCAGGCTGCAGGAGGAGTG	Homologous recombination	pfastbac-VIP1R(31-437)-LgBiT
VIP1R(31-437) Reverse	TTCGAGTGTGAAGACCAGCATGGAAACCTGCGTGC		
Linear pfastbac Forward	GTCTTCACACTCGAAGATTTCGTTG		
Linear pfastbac Reverse	GTGATGGTGATGGTGATGGTGATG		
VIP1R Forward	GGTGTCCACTCCGAGGCCAGGCTGCAGGAGGAGTGTG	Homologous recombination	pcDNA6.0-VIP1R mutants
VIP1R(31-437)-LgBiT Reverse	GAAGGGCCCTCTAGATTAGCTGTTGATGGTTACTCGG		
VIP1R(31-437) Reverse	GGGCCCTCTAGATTACAGCATGGAAACCTG		
VIP1R (31-457) Reverse	GGGCCCTCTAGATTAGACCAGGGAGACTTCGGC		
Linear pcDNA Forward	TAATCTAGAGGGCCCTTC		
Linear pcDNA Reverse	CTCGGAGTGGACACCTGTG		
Q135A Forward	GAGCAGGCCACCATGTTCTACGGTTC	Site-directed mutagenesis	pcDNA6.0-VIP1R(31-457, Q135A)
Q135A Reverse	CATGGTGGCCTGCTCATCCAAACTC		
T136A Forward	CAGCAGGCCATGTTCTACGGTTC		pcDNA6.0-VIP1R(31-457, T136A)
T136A Reverse	GAACATGGCCTGCTGCTCATCCAAAC		
Y139A Forward	ATGTTCGCCGGTTCTGTGAAGACC		pcDNA6.0-VIP1R(31-457, Y139A)
Y139A Reverse	AGAACCGGCGAACATGGTCTGCTGC		
V142A Forward	GGTTCTGCCAAGACCGGCTACACC		pcDNA6.0-VIP1R(31-457, V142A)
V142A Reverse	GGTCTTGGCAGAACCGTAGAACATGG		
Y146A Forward	ACCGGCGCCACCATCGGCTACGGC		pcDNA6.0-VIP1R(31-457, Y146A)
Y146A Reverse	GATGGTGGCGCCGGTCTTCACAGAAC		
R188A Forward	TTGGCCGCCTTCGACAGCGGGGAG		pcDNA6.0-VIP1R(31-457, R188A)
R188A Reverse	GTCGAAGGCGGCCAAGTCTTTGATG		
L199A Forward	GTCTTTGCCCAATATTGTGTCATGGC		pcDNA6.0-VIP1R(31-457, L199A)
L199A Reverse	ATATTGGGCAAAGACCATGGCTGC		
F222A Forward	TTTTTCGCCTATTGTGTCATGGCTAAC		pcDNA6.0-VIP1R(31-457, F222A)
F222A Reverse	ACAATAGGCGAAAAAGACCATGGCTG		
Q223A Forward	TATTGTGCCATGGCTAACTTCTTC		pcDNA6.0-VIP1R(31-457, Q223A)
Q223A Reverse	AGCCATGGCACAATATTGGAAAAAGAC		
V226A Forward	GCTAACGCCTTCTGGCTGCTGGTGG		pcDNA6.0-VIP1R(31-457, V226A)
V226A Reverse	CCAGAAGGCGTTAGCCATGACACAATATTGG		
F230A Forward	GACACCGCCAACTCCTCACTGTGGTG		pcDNA6.0-VIP1R(31-457, F230A)
F230A Reverse	GGAGTTGGCGGTGTCCCAGCACCC		
I289A Forward	TCACTGGCCTGGATCATAAAGGGCCCC		pcDNA6.0-VIP1R(31-457, I289A)
I289A Reverse	GATCCAGGCCAGTGAGGAGTTGATG		
W294A Forward	GGCCCCGCCCTCACCTCCATCTTGG		pcDNA6.0-VIP1R(31-457, W294A)
W294A Reverse	GGTGAGGGCGGGGCCCTTTATGATCC		
I301A Forward	GTGAAGGCCGTCTTTGAGCTCGTCG		pcDNA6.0-VIP1R(31-457, I301A)
I301A Reverse	AAAGACGGCCTTCACTTCAGGCTTAAAATTG		
M370A Forward	TTTGAGGCCGTCGTGGGGTCTTTCCAG		pcDNA6.0-VIP1R(31-457, M370A)
M370A Reverse	CACGACGGCCTCAAAGACCATCTTC		
L374A Forward	ATCCTGGCCGCTGCCGCTGTCTTCATC		pcDNA6.0-VIP1R(31-457, L374A)
L374A Reverse	GGCAGCGGCCAGGATGAAGGATATGAAGAGGTG		
