# Supplementary material for: Cryo-EM structure of an activated VIP1 receptor-G protein complex revealed by a NanoBiT tethering strategy
Source: Nat Commun. 2020 Aug 17;11:4121. doi: 10.1038/s41467-020-17933-8 (PMC7431577; doi:10.1038/s41467-020-17933-8)
Supplement: Supplementary file 3 — Reporting Summary [file 41467_2020_17933_MOESM3_ESM.pdf]

Corresponding author(s): Yi Jiang
Last updated by author(s): Jul 16, 2020

Reporting Summary

Nature Research wishes to improve the reproducibility of the work that we publish. This form provides structure for consistency and transparency in reporting. For further information on Nature Research policies, see our Editorial Policies and the Editorial Policy Checklist.

Statistics

For all statistical analyses, confirm that the following items are present in the figure legend, table legend, main text, or Methods section.

n/a	Confirmed	
☐	☒	The exact sample size (*n*) for each experimental group/condition, given as a discrete number and unit of measurement
☐	☒	A statement on whether measurements were taken from distinct samples or whether the same sample was measured repeatedly
☐	☒	The statistical test(s) used AND whether they are one- or two-sided<br>*Only common tests should be described solely by name; describe more complex techniques in the Methods section.*
☒	☐	A description of all covariates tested
☒	☐	A description of any assumptions or corrections, such as tests of normality and adjustment for multiple comparisons
☐	☒	A full description of the statistical parameters including central tendency (e.g. means) or other basic estimates (e.g. regression coefficient) AND variation (e.g. standard deviation) or associated estimates of uncertainty (e.g. confidence intervals)
☐	☒	For null hypothesis testing, the test statistic (e.g. *F*, *t*, *r*) with confidence intervals, effect sizes, degrees of freedom and *P* value noted<br>*Give P values as exact values whenever suitable.*
☒	☐	For Bayesian analysis, information on the choice of priors and Markov chain Monte Carlo settings
☒	☐	For hierarchical and complex designs, identification of the appropriate level for tests and full reporting of outcomes
☒	☐	Estimates of effect sizes (e.g. Cohen's *d*, Pearson's *r*), indicating how they were calculated

Our web collection on statistics for biologists contains articles on many of the points above.

Software and code

Policy information about availability of computer code

Data collection	FEI TEM user interface, SerialEM
Data analysis	MotionCor 2.1, Gctf v1.06, RELION 3.0, Chimera 1.13, Phenix 1.13_2998, Coot 0.8.9.2, MolProbity, PyMOL 2.3.2, MOLE 2.5

For manuscripts utilizing custom algorithms or software that are central to the research but not yet described in published literature, software must be made available to editors and reviewers. We strongly encourage code deposition in a community repository (e.g. GitHub). See the Nature Research guidelines for submitting code & software for further information.

Data

Policy information about availability of data

All manuscripts must include a data availability statement. This statement should provide the following information, where applicable:

- Accession codes, unique identifiers, or web links for publicly available datasets
- A list of figures that have associated raw data
- A description of any restrictions on data availability

Density maps and structure coordinates have been deposited to the Electron Microscopy Database and the Protein Data Bank with accession number of EMD-21249, PDB ID 6VN7 for the PACAP27-VIP1R-Gs complex.

Field-specific reporting

Please select the one below that is the best fit for your research. If you are not sure, read the appropriate sections before making your selection.

☒ Life sciences ☐ Behavioural & social sciences ☐ Ecological, evolutionary & environmental sciences

For a reference copy of the document with all sections, see nature.com/documents/nr-reporting-summary-flat.pdf

Life sciences study design

All studies must disclose on these points even when the disclosure is negative.

Sample size	Cryo-EM images were collected until the resolution and 3D reconstruction converges.
Data exclusions	No data were excluded from the analysis apart badly picked or low resolution particles from EM data.
Replication	Experimental findings were reliably reproduced.
Randomization	Randomization was not relevant for this study, as data were collected automatically and did not involve choosing.
Blinding	Blinding was not relevant to the study, since all the data were collected automatically.

Reporting for specific materials, systems and methods

We require information from authors about some types of materials, experimental systems and methods used in many studies. Here, indicate whether each material, system or method listed is relevant to your study. If you are not sure if a list item applies to your research, read the appropriate section before selecting a response.

Materials & experimental systems

n/a	Involved in the study	
☐	☒	Antibodies
☐	☒	Eukaryotic cell lines
☒	☐	Palaeontology and archaeology
☒	☐	Animals and other organisms
☒	☐	Human research participants
☒	☐	Clinical data
☒	☐	Dual use research of concern

Methods

n/a	Involved in the study	
☒	☐	ChIP-seq
☐	☒	Flow cytometry
☒	☐	MRI-based neuroimaging

Antibodies

Antibodies used	Monoclonal ANTI-FLAG® M2 antibody (Sigma, No. F3165), Goat anti-Mouse IgG (H+L) Highly Cross-Adsorbed Secondary Antibody, Alexa Fluor 488 (Invitrogen, No.A-11029)
Validation	Monoclonal ANTI-FLAG® M2 antibody: https://www.sigmaaldrich.com/catalog/product/sigma/f3165?lang=en&region=US<br>Goat anti-Mouse IgG (H+L) Highly Cross-Adsorbed Secondary Antibody, Alexa Fluor 488: https://www.thermofisher.com/cn/en/antibody/product/Goat-anti-Mouse-IgG-H-L-Highly-Cross-Adsorbed-Secondary-Antibody-Polyclonal/A-11029<br>All antibodies used are commercially purchased and have been validated by the vendors. Validation data are available from the respective vendor's respective websites.

Eukaryotic cell lines

Policy information about cell lines

Cell line source(s)	Mammalian CHO-K1 cells: ATCC no. CCL-61, Insect cells: Invitrogen (Sf9)
Authentication	Used as expression strains only, independent verification after purchase not required.
Mycoplasma contamination	The above cell lines were negative for Mycoplasma contamination.
Commonly misidentified lines (See ICLAC register)	No commonly misidentified cell lines were used.

Flow Cytometry

Plots

Confirm that:

- [ ] The axis labels state the marker and fluorochrome used (e.g. CD4-FITC).
- [ ] The axis scales are clearly visible. Include numbers along axes only for bottom left plot of group (a 'group' is an analysis of identical markers).
- [ ] All plots are contour plots with outliers or pseudocolor plots.
- [ ] A numerical value for number of cells or percentage (with statistics) is provided.

Methodology

Sample preparation	Sample preparation listed in Methods
Instrument	BD Accuri C6 (BD Biosciences)
Software	BD Accuri C6 software (version 1.0.264.21)
Cell population abundance	Approximately 10,000 cellular events were collected and the total fluorescence intensity of positive expression cell population was calculated.
Gating strategy	Gating was determined by the Alexa-488 fluorescence intensity to differentiate positive cells and all other cells.

- [x] Tick this box to confirm that a figure exemplifying the gating strategy is provided in the Supplementary Information.
