# Supplementary material for: Cryo-EM structure of an activated VIP1 receptor-G protein complex revealed by a NanoBiT tethering strategy
Source: Nat Commun. 2020 Aug 17;11:4121. doi: 10.1038/s41467-020-17933-8 (PMC7431577; doi:10.1038/s41467-020-17933-8)
Supplement: Supplementary file 2 — Peer Review File [file 41467_2020_17933_MOESM2_ESM.docx]

Peer Review Information

**Journal:** Nature Communications

**Manuscript title:** Cryo-EM structure of an activated VIP1 receptor-G protein complex revealed by a NanoBiT tethering strategy

**Corresponding author name(s):** Dr Yi Jang

**Editorial notes:**

**Transferred manuscripts**

This manuscript has been previously reviewed at another journal that is not operating a transparent peer review scheme. This document only contains reviewer comments and rebuttal letters for versions considered at *Nature Communications*.

**Reviewer comments & decisions:**

**Reviewer comments, first version:**

Reviewer #1 (Remarks to the Author):

In the manuscript by Duan et al, the authors describe the novel application of a protein tethering method to help determine the structure of a complex of the vasoactive intestinal polypeptide (VIP) receptor, VIP1-R, bound to the Gs heterotrimer, in an agonist (PACAP)-bound and nucleotide-free form. The tethering strategy, utilizing the NanoBiT™ system, enhances the interaction between Gβ of the Gs heterotrimer and the C-terminus of the VIP1-R. Dynamic light scattering and negative stain EM analyses suggest that using the tethering method increases the monodispersity of the complex and yield more homogeneous particles. The enhanced homogeneity facilitated single particle analysis by cryoEM, yielding a relativley higher resolution structure of the VIP1-R-Gs complex than the more typical cryoEM data on R-G complexes. The novel structure reveals the hallmark properties of an activated GPCR bound to a nucleotide-free G protein complex. Some of the structural similarities of the G protein may be influenced by the use of G protein nanobody, Nb35. All R-G complexes structures with Gs published to date have taken advantage of Nb35. What is novel about the structure, of course, is the resolution of the interaction between the neuropeptide agonist, pituitary adenylate cyclase-activating peptide (PACAP), and VIP1-R. However, the more interesting technological advancement described in this manuscript may the use of the NanoBiT tethering strategy, as it appears to be transferable to other receptor-G protein systems.

In summary, the manuscript by Duan et al describes a novel strategy to obtain a novel structure of an R-G complex, and should be of significant interest to the G protein-coupled receptor field. There are several issues, however, that need to be addressed.

Comments:

1. While the study is very interesting the description of work is rather poorly descrdibed and edited and detracts away from the important message that the authors are trying to convey. In several occasions it was not clear what point the authors were trying to support. For example, in lines 148-150 the authors state, "In addition, the extracellular domain (ECD) of VIP1R is invisible in the density map because of its highly dynamic conformation". This might be rewritten as "the extracellular domain (ECD) of VIP1R was not resolvable with this limited data set, perhaps reflecting its highly dynamic and conformationally flexible property when bound to PACAP27".

In lines 204-206, the authors state "The structural hallmark of class B GPCR activation is the pronounced outward shift of TM6, which is accompanied by formation of a sharp kink in the middle of the TM6 induced by ligand binding". While this statement is more or less true it gives the impression that only class B GPCRs display a change in the TM6 structure upon receptor activation. It is quite clear from the structures of members of several GPCR families reported thus far reveal conformational changes in TM6 associated with receptor activation. In addition, it should be clarified that TM6 movement in GPCRs may be 'stabilized by agonist binding', and not merely 'induced by ligand binding'. It should also be noted that several biophysical studies on different GPCRs suggest that G protein binding also helps to stabilize this conformational change.

Likewise, in lines 242-244 the authors state "Taken together, despite the different sequence and physicochemical environment of VIP1R, VIP1R shares a common activation mechanism with other class B GPCRs, which is characterized by ligand-induced conformational changes in the receptor helix bundle". Is this a class B GPCR-specific change, or is it shared among all classes of GPCRs ?

In lines 240-242 the authors state, "The HETx and TM2-6-7-helix 8 polar networks are, therefore, required for maintaining an inactive conformation, and the breakage of these polar networks represents active conformations of VIP1R". Is it really known that the HETx and TM2-6-7-helix 8 polar networks are required for the inactive conformation of the VIP1-R, or just speculation ? Furthermore, is it known that disruption of these networks is associated with receptor activation ? This needs to be clarified by the authors.

In lines 282-283 the authors state "This is consistent with the fact that the VIP1R-Gs complex is not sufficiently stable for cryo-EM studies without NanoBiT tethering", after describing the differences with the PTH1R-Gs complex. It is not clear how application of the NanoBiT tether could influence the this.

In lines 259-260 the authors refer to a structural alignment of the VIP1R-Gs with the structures of other class B receptor-G protein complexes. Are the 'other' structures described in the comparison novel structures or are they previously reported ? Although better described in the supplement, this should be clearly stated in the manuscript body.

2. It would be helpful to the field if the authors could present some data, or at least comment on the composition and characteristics of the linkers used in the tethering constructs. Are there optimal lengths that may be revealed by DLS or negative stain EM ?

3. In lines 263-264 the authors state "The Gs proteins themselves do not show significant conformational differences". It should be stated by the authors that the structural similarities in the Gs heterotrimer may be influenced by the use of Nb35, which has been used in all the structures of Gs-coupled receptor complexes reported thus far.

4. It would be helpful for the field to know if GTPγS or GDP-(AlF4-) can disrupt the complex or whether the energetic contributions of the NanoBiT tether are insurmountable with G protein activation.

5. In lines 49-51 the authors state "The NanoBiT tethering technology has been demonstrated to be an effective tool for structural studies of unstable GPCR-G protein or other complex systems". Some reference to their preliminary data on the application of this tethering method to the CCR7-Gi complex should be stated here. Otherwise the this introductory statement should simply state that the methodology may help to provide structural information of unstable complexes.

Minor:

The authors need to describe G protein as the Gs heterotrimer and not merely the 'Gs protein'.

The authors include more details about the negative staining conditions (eg. pH, etc).


Reviewer #2 (Remarks to the Author):

In this manuscript, Duan et al. solved a cryo-EM structure of the PACAP-VIP1R-Gs complex and described a mechanism of VIP1R activation via ligand binding. While the VIP1R structure is novel, it lacks the ECD of the receptor, which is another important region to facilitate ligand binding, presumably due to its flexibility. The authors utilized the high-affinity complementation of the Nanobit pair resulting in improvement of GPCR-G protein stability, and showed that the strategy works in another GPCR, CCR7, as well.

-Major comments
The authors should more carefully compare the solved structure with previous studies on ligand alanine scanning (e.g., Igarashi et al. JPET 301, 37 2002) and describe how well (or poorly in some occasions) the current structure explains such previous mutagenesis studies.

Is a density of the Nanobit in the structure visible? If not, why is it so despite the fact that the Nanobit pair stabilizes the VIP1R-Gs complex?

In Fig.1b and Fig.S3a, the authors should indicate molecular markers or chromatogram of a GPCR monomer (e.g., FSEC) so that the peak corresponds to the complex and is distinguishable from a dispersed sample.

In Fig.1c and Fig.S3c, what does the y-axis mean? The reviewer is confused about whether the y-axis reflects a frequency of each radius population. If so, why do the total areas (sum of the bars) differ between the two panels? Is it more informative by showing a ratio of the monomer peak area to that of the aggregation peak area?

The manuscript will be improved by including recent publications on PACAP-PAC1-Gs structures (Ligand et al. Mol Cell 2020; Wang et al. Cell Res 2020; Kobayashi et al. BioRxiv). Specifically, the reviewer recommends that the authors compare a mode of PACAP recognition between VIP1R and PAC1 and further discuss how a ligand selectivity (VIP1R, but not PAC1, binds VIP) is explained by the structures.

The authors should include a method for the Nanobit G-protein coupling assay. Is this a previously validated method? The reviewer recommends that a representative concentration-response be included in the supplementary figure so that readers can evaluate quality of the assays (signal window, signal-to-background level, etc.). How do the authors normalize expression levels of VIP1R mutants? Why did the authors used the Nanobit G-protein coupling assay over the cAMP assay or a ligand binding/competition assay, the latter of which measures binding affinity more directly?

In Fig.S1, please show cAMP response of mock-transfected cells. The reviewer happened to have seen that HEK293 cells respond to PACAP via its endogenous receptor. It is unclear how the y-axis in Fig.S1 was calculated (e.g., cAMP mole or LANCE count?; normalized to VIP1R-WT or each construct?). Since the authors used the LANCE cAMP assay, in which quantification is possible by using a cAMP standard, the reviewer recommends that unit of y-axis be absolute cAMP level (mole per well).

-Minor comment
Although small fragments were named as 114 and 86 for low-affinity and high-affinity peptides, respectively, in Dixon et al. 2016, the author suggests that the peptides be labeled as SmBiT and HiBiT, respectively, throughout the manuscript in order to match Promega's recent naming. In the author's understanding, SmBiT refers to the peptide 114.


Reviewer #3 (Remarks to the Author):

This manuscript reports a 3.2 A cryo-EM structure of VIPR1-G protein in complex with an activating peptide PACAP27. The authors creatively included a newly developed NanoBiT method to strengthen the binding interface of VIPR1 and Gs protein, which provided an option for future structural analysis between GPCR and downstream G protein or other adaptors. The structure is solid, and resembles previous determined class B complex structures, and the structural mechanism for how peptide triggers the conformational change and receptor activation is well analyzed and described. The paper is well written too.
Main questions:
1. The ECD is surprisingly missing in recent cryo-EM structures, which looks odd as the well accepted mode suggests binding of peptide first to the ECD and then to the TMD pocket, and the helical structure of the hormone peptide is supposed to be quite rigid to stabilize the orientation of the bound ECD. Furthermore, there are literatures suggesting additional disulfide linkage between the ECD N-terminus and the ECL1 (e.g., vilardaga, 1997), which may further strengthen the orientation of ECD. The recently published PAC1 complex structure may give some clue about this by showing continued density of the ECD N-terminus and ECL1. Is it possible for the authors to revisit the original EM data of VIPR1 to see if there are more clues, or comments on this issue?
2. Since the close analog of VIPR1, PAC1R, was published recently in Molecular Cell, can the author compare further with that structure? Or at least, what may be the mechanism that PACAP can recognize both receptors but VIP binds mainly with VIPR1/2?
Minor points:
1. Why use PACAP for the complex study? Was the VIP also tested for the complex formation?
2. It is brilliant to use the NanoBiT method for the complex formation, but it is also suspected that the NanoBiT would need the peptide from the gamma subunit for the exact folding. So I was wondering when expressed alone, are the expression levels of VIPR with and without the NanoBiT similar? Is the density of NanoBit visible in the final model?


Reviewer #4 (Remarks to the Author):

In this paper, the authors utilised NanoBiT technology reported by Dixon et al., 2016 to enhance stability of GPCR complex for cryo-EM. For this purpose, they have chosen the peptide 86 from the manuscript, which has the highest binding affinity (Kd ~900pM). This improved the stability of two GPCR G protein complexes and could be useful for other more challenging complex. Using this approach, they have improved the biochemistry and solved the structure of VIPR-Gs complex bound to PACAP-27. Notably, this is a novel structure of a class B GPCR that has not been previously solved. Although the ECD of the receptor was poorly resolved, there are good densities for 7TM, G proteins and N-term of peptide, in accord with reported 3.2A resolution.

Major comments:
- Constructs of receptor (WT, truncated, truncated + LgBiT) were validated; however, there was no validation for Gbeta construct fused with SmBiT.
- Method for NanoBit assay performed for evaluating G protein coupling activities (Table S4) was not described
- Methods for complex formation of CCR7/Gi, scFv16 generation were not described.
- Lack of detail in construct generated for VIPR, was there a linker between C-term of the receptor and LgBiT? It was not mentioned that there was no density observed for the LgBiT and SmBiT as they should be present in the sample. Was there any density observed for the NanoBiT in the intermediate cryo-EM map during processing?
- The density of receptor ECD was almost invisible in the cryo-EM map, ECD of class B receptor has a vital role in peptide binding and there have been differences in the orientation of ECD relative to the receptor core observed for different class B receptors. Has the author tried to alternative method i.e creating a mask on the receptor only to improve the ECD region?
- Density for the peptide appeared to be too poor to be confidently modelled after residue 15 (at least in the map supplied). Interactions beyond residue 15 in the peptide should not be discussed.


Minor comments:
- Negative stain micrograph in Figure 1d has poor resolution, I failed to observe any differences between the two.
- Typo - Line 390, “barelly”
- While SmBiT fused with chain B was included, LgBiT sequence fused with chain R was not included in the PDB report.
- Lane 79, ref 14 – this review did not discuss impact of cryo-EM on GPCR structural biology.

**Author rebuttal, first version:**

**Reviewer #1:**

In the manuscript by Duan et al, the authors describe the novel application of a protein tethering method to help determine the structure of a complex of the vasoactive intestinal polypeptide (VIP) receptor, VIP1-R, bound to the Gs heterotrimer, in an agonist (PACAP)-bound and nucleotide-free form. The tethering strategy, utilizing the NanoBiT™ system, enhances the interaction between Gβ of the Gs heterotrimer and the C-terminus of the VIP1-R. Dynamic light scattering and negative stain EM analyses suggest that using the tethering method increases the monodispersity of the complex and yield more homogeneous particles. The enhanced homogeneity facilitated single particle analysis by cryoEM, yielding a relativley higher resolution structure of the VIP1-R-Gs complex than the more typical cryoEM data on R-G complexes. The novel structure reveals the hallmark properties of an activated GPCR bound to a nucleotide-free G protein complex. Some of the structural similarities of the G protein may be influenced by the use of G protein nanobody, Nb35. All R-G complexes structures with Gs published to date have taken advantage of Nb35. What is novel about the structure, of course, is the resolution of the interaction between the neuropeptide agonist, pituitary adenylate cyclase-activating peptide (PACAP), and VIP1-R. However, the more interesting technological advancement described in this manuscript may the use of the NanoBiT tethering strategy, as it appears to be transferable to other receptor-G protein systems.

In summary, the manuscript by Duan et al describes a novel strategy to obtain a novel structure of an R-G complex, and should be of significant interest to the G protein-coupled receptor field. There are several issues, however, that need to be addressed.

We thank the Reviewer for the positive comment.

Comments:

1. While the study is very interesting the description of work is rather poorly described and edited and detracts away from the important message that the authors are trying to convey. In several occasions it was not clear what point the authors were trying to support. For example, in lines 148-150 the authors state, "In addition, the extracellular domain (ECD) of VIP1R is invisible in the density map because of its highly dynamic conformation". This might be rewritten as "the extracellular domain (ECD) of VIP1R was not resolvable with this limited data set, perhaps reflecting its highly dynamic and conformationally flexible property when bound to PACAP27".

Response: We agree with the Reviewer’s comments and have re-written this sentence and other sentences throughout the manuscript text. Specifically, this sentence was rewritten as below: "In addition, the extracellular domain (ECD) of VIP1R was not resolvable with this limited data set, perhaps reflecting its highly dynamic and conformationally flexible property when bound to PACAP27. This is consistent with the highly dynamic nature of ECD in class B GPCRs when bound to activating ligands as the ECD structures were not well resolved in most of other active class B GPCR-G protein complexes.”

In lines 204-206, the authors state "The structural hallmark of class B GPCR activation is the pronounced outward shift of TM6, which is accompanied by formation of a sharp kink in the middle of the TM6 induced by ligand binding". While this statement is more or less true it gives the impression that only class B GPCRs display a change in the TM6 structure upon receptor activation. It is quite clear from the structures of members of several GPCR families reported thus far reveal conformational changes in TM6 associated with receptor activation. In addition, it should be clarified that TM6 movement in GPCRs may be 'stabilized by agonist binding', and not merely 'induced by ligand binding'. It should also be noted that several biophysical studies on different GPCRs suggest that G protein binding also helps to stabilize this conformational change.

Response: We agree the Reviewer’s comment that the outward movement of TM6 is not limited to class B GPCRs, except that the outward movement of TM6 is much more pronounced in class B GPCRs than class A GPCRs. The sentence has been rewritten to "The structural hallmark of class B GPCR activation is the much more pronounced outward shift of TM6 than that in class A GPCRs, which is accompanied by formation of a sharp kink in the middle of the TM6 induced and stabilized by ligand binding".

Likewise, in lines 242-244 the authors state "Taken together, despite the different sequence and physicochemical environment of VIP1R, VIP1R shares a common activation mechanism with other class B GPCRs, which is characterized by ligand-induced conformational changes in the receptor helix bundle". Is this a class B GPCR-specific change, or is it shared among all classes of GPCRs?

Response: We thank the Reviewer for the comment. This is indeed a general activation mechanism shared among all GPCRs, however, the specific residues involved in receptor activation and G protein coupling are quite conserved among class B GPCRs but very different from class A GPCRs. We have rewritten the sentence to ”Taken together, despite the different sequence and physicochemical environment of VIP1R in ligand binding pocket, VIP1R shares a common activation mechanism with other class B GPCRs, which is characterized by a set of conserved residues involved in ligand-induced conformational changes in the receptor helix bundle as well as residues involved in G protein coupling".

In lines 240-242 the authors state, "The HETx and TM2-6-7-helix 8 polar networks are, therefore, required for maintaining an inactive conformation, and the breakage of these polar networks represents active conformations of VIP1R". Is it really known that the HETx and TM2-6-7-helix 8 polar networks are required for the inactive conformation of the VIP1-R, or just speculation? Furthermore, is it known that disruption of these networks is associated with receptor activation? This needs to be clarified by the authors.

Response: Because the inactive VIP1R structure is not available, the statement that the HETx and TM2-6-7-helix 8 polar networks are required for the inactive conformation of the VIP1-R is speculative. In addition, the x in the HETx motif is a tyrosine except a phenylalanine in calcitonin-like receptor (CALRL), thus we rename it as the HETY motif in the text. This part of text has been rewritten as below. “VIP1R contains the conserved HETY motif, which is known to mediated inter-helix interactions of TM2-6-7-helix 8 polar networks in GCGR, PTH1R, and CRFR1. Disruption of this inter-helix interactions has resulted in constitutively active receptors. We therefore speculate that TM2-3-6-7 polar networks may also be required for maintaining an inactive conformation and the breakage of these polar networks may represent the active conformations of VIP1R. Indeed, mutations that disrupt this polar network in VIP1R has resulted in constitutively active receptor.” The mutation data has been published by our group earlier in a 2017 JBC paper ([*J Biol Chem*](https://www.ncbi.nlm.nih.gov/pmc/articles/PMC5473240/). 2017 Jun 16; 292(24): 9865–9881) and the data is attached below.

Figure. Mutations of the residues in HETY motif induce the constitutive activation of VIP1R.

In lines 282-283 the authors state "This is consistent with the fact that the VIP1R-Gs complex is not sufficiently stable for cryo-EM studies without NanoBiT tethering", after describing the differences with the PTH1R-Gs complex. It is not clear how application of the NanoBiT tether could influence the this.

Response: Under our experimental conditions, PTH1R can form a stable Gs complex while VIP1R cannot. After structure determination, we found that VIP1R has a smaller Gs binding interface than that of PTH1R. The tethered NanoBiT probably provide additional stabilization between VIP1R and Gs protein to partly overcome the fewer interactions in this smaller Gαs-buried surface of VIP1R-Gs complex compared to other class B GPCRs.

In lines 259-260 the authors refer to a structural alignment of the VIP1R-Gs with the structures of other class B receptor-G protein complexes. Are the 'other' structures described in the comparison novel structures or are they previously reported? Although better described in the supplement, this should be clearly stated in the manuscript body.

Response: We thank the Reviewer for the comment. The “other” structures stand for all class B GPCRs-Gs complexes with previously reported structures. We have rewritten this sentence to make the statement more clearly.

2. It would be helpful to the field if the authors could present some data, or at least comment on the composition and characteristics of the linkers used in the tethering constructs. Are there optimal lengths that may be revealed by DLS or negative stain EM?

Response: We thank the Reviewer for the comment. The flexible C-terminus of VIP1R serves as the natural linker to connect LgBiT directly. The wild type VIP1R and different lengths of C-terminus truncated VIP1R at L437, G424, and K417 were screened for assembly of the complex. When the receptor was truncated to L437, the components can be assembled into the VIP1R(31-437)-VIP1R-Gs complex with a equal proportion, suggesting a better assembly efficacy for the complex. Corresponding comments about have been added in text.

3. In lines 263-264 the authors state "The Gs proteins themselves do not show significant conformational differences". It should be stated by the authors that the structural similarities in the Gs heterotrimer may be influenced by the use of Nb35, which has been used in all the structures of Gs-coupled receptor complexes reported thus far.

Response: We agree with the Reviewer and have added that statement in the text.

4. It would be helpful for the field to know if GTPγS or GDP-(AlF4-) can disrupt the complex or whether the energetic contributions of the NanoBiT tether are insurmountable with G protein activation.

Response: We thank the Reviewer for the constructive advice. We use the highest affinity binding small BIT, termed HiBIT, which has pM affinity to the LgBiT, instead of the lowest affinity SmBiT, used in normal NanoBIT complementary assays. The interaction of HiBiT to LgBiT is stable and independent of the status of G proteins. In the case of SmBiT, we expect that it would be affected by the presence of GTPγS or GDP-(AlF4-).

5. In lines 49-51 the authors state "The NanoBiT tethering technology has been demonstrated to be an effective tool for structural studies of unstable GPCR-G protein or other complex systems". Some reference to their preliminary data on the application of this tethering method to the CCR7-Gi complex should be stated here. Otherwise the this introductory statement should simply state that the methodology may help to provide structural information of unstable complexes.

Response: We thank the Reviewer for the suggestion. We have rewrite this sentence as “The methodology of the NanoBiT tethering may help to provide structural information of unstable complexes” as the Reviewer suggested.

Minor:

The authors need to describe G protein as the Gs heterotrimer and not merely the 'Gs protein'.

Response: Thank you for pointing out our negligence. We have changed “Gs protein” to “Gs heterotrimer” in our revised manuscript.

The authors include more details about the negative staining conditions (eg. pH, etc).

Response: We thank the Reviewer for the suggestion and have included more details about the negative staining conditions in the methods section.

**Reviewer #2:**

In this manuscript, Duan et al. solved a cryo-EM structure of the PACAP-VIP1R-Gs complex and described a mechanism of VIP1R activation via ligand binding. While the VIP1R structure is novel, it lacks the ECD of the receptor, which is another important region to facilitate ligand binding, presumably due to its flexibility. The authors utilized the high-affinity complementation of the Nanobit pair resulting in improvement of GPCR-G protein stability, and showed that the strategy works in another GPCR, CCR7, as well.

We thank the Reviewer for the comment.

Major comments

The authors should more carefully compare the solved structure with previous studies on ligand alanine scanning (e.g., Igarashi et al. JPET 301, 37 2002) and describe how well (or poorly in some occasions) the current structure explains such previous mutagenesis studies.

Response: We thank the Reviewer for the advice. We have added the discussion to compare the potential recognition modes of VIP1R by PACAP and VIP in “PACAP recognition by VIP1R and PAC1R” section.

Is a density of the Nanobit in the structure visible? If not, why is it so despite the fact that the Nanobit pair stabilizes the VIP1R-Gs complex?

Response: The density of the NanoBiT is invisible in our structure. We suspect that the NanoBiT can increase the local concentration of Gs hetertrimer near VIP1R, and also make the Gs hetertrimer not easily dissociated from the receptor. Compared with the VIP1R-Gs protein complex, the NanoBiT is relatively flexible because of the existence of the linker between LgBiT and the receptor. The stabilization mode of NanoBiT is different from the antibodies which bind against the antigen directly. So the density of antibodies is visible in most of GPCR complex structures.

In Fig.1b and Fig.S3a, the authors should indicate molecular markers or chromatogram of a GPCR monomer (e.g., FSEC) so that the peak corresponds to the complex and is distinguishable from a dispersed sample.

Response: We thank the Reviewer for his/her valuable advice. We have labeled molecular markers in these figures in our revised manuscript.

In Fig.1c and Fig.S3c, what does the y-axis mean? The Reviewer is confused about whether the y-axis reflects a frequency of each radius population. If so, why do the total areas (sum of the bars) differ between the two panels? Is it more informative by showing a ratio of the monomer peak area to that of the aggregation peak area?

Response: We apologize for any confusion. The y-axis refers to the relative amount of light scattered by each population (%intensity), which reflects a frequency of each radius population as the Reviewer suggested. For the NanoBiT-tethering sample, its sample particles of monomer have a relatively higher %intensity and narrower distribution than the wild type one. Accordingly, the total areas in two panels should be identical. The peak distributions of these two samples indicate that the NanoBiT-tethering sample is more homogeneous than the wild type sample.

Additionally, the values of %Intensity stands for the percentage of monomer in a total sample. We agree with the Reviewer’s comment and change the values to the ratio of the monomer to the aggregation which is more informative.

The manuscript will be improved by including recent publications on PACAP-PAC1-Gs structures (Ligand et al. Mol Cell 2020; Wang et al. Cell Res 2020; Kobayashi et al. BioRxiv). Specifically, the Reviewer recommends that the authors compare a mode of PACAP recognition between VIP1R and PAC1 and further discuss how a ligand selectivity (VIP1R, but not PAC1, binds VIP) is explained by the structures.

Response: We thank the Reviewer for his/her invaluable advice. The structural comparison of PACAP27-VIP1R-Gs with and PACAP38-PAC1R-Gs complex (PDB code: 6P9Y, Liang et al. *Mol Cell* 2020) has been performed to clarify the recognition mechanism of these two receptors by PACAP. The corresponding text has been added to the “PACAP recognition by VIP1R and PAC1R” section. The figures on the structural comparison of these two PACAP-receptor binding pockets were also added in Fig. 3d-h. Additionally, we have added the discussion in the revised manuscript to provide a potential explanation of VIP selectivity for VIP1R over PAC1R (Supplementary Fig. 7).

The authors should include a method for the Nanobit G-protein coupling assay. Is this a previously validated method? The Reviewer recommends that a representative concentration-response be included in the supplementary figure so that readers can evaluate quality of the assays (signal window, signal-to-background level, etc.). How do the authors normalize expression levels of VIP1R mutants? Why did the authors used the Nanobit G-protein coupling assay over the cAMP assay or a ligand binding/competition assay, the latter of which measures binding affinity more directly?

Response: We apologized for our negligence. The method in Table S4 should be the TR-FRET assay which measures the cAMP accumulation, but not the NanoBiT assay, which evaluates G protein coupling. It has been revised to correct description in our revised manuscript.

In Fig.S1, please show cAMP response of mock-transfected cells. The Reviewer happened to have seen that HEK293 cells respond to PACAP via its endogenous receptor. It is unclear how the y-axis in Fig.S1 was calculated (e.g., cAMP mole or LANCE count? normalized to VIP1R-WT or each construct?). Since the authors used the LANCE cAMP assay, in which quantification is possible by using a cAMP standard, the reviewer recommends that unit of y-axis be absolute cAMP level (mole per well).

Response: Thanks for your constructive advice. The Reviewer is correct. The mock 293T cells used in our manuscript show a strong response to PACAP27 with the pEC50 at 9.75. We have attached the result in the following figure. So, we have reperformed all the cAMP accumulation assay using the CHO-K1 cells, which have a much lower response to PACAP27. All the cAMP accumulation data have been updated in the revised manuscript (Fig.S1 and Table S4).

Additionally, we used the relative cAMP signals but not the absolute cAMP amount to calculate pEC50 of each group of receptor truncations in our LANCE cAMP assay. So, the y-axis in Fig.S1 stands for the cAMP accumulation signal normalized to VIP1R (WT). The activation analysis of GPCRs based on relative cAMP signals has also been widely used in GPCR functional analysis ([*Mol Cell*.](https://www.ncbi.nlm.nih.gov/pubmed/32004470) 2020 Feb 6;77(3):669-680), and the values of pEC50s should also be accurate.

Figure. Effects of PACAP27 on activation of mock and wild type VIP1R-transfected 293T cells. cAMP accumulation signals were normalized to the mock group.

Minor comment

Although small fragments were named as 114 and 86 for low-affinity and high-affinity peptides, respectively, in Dixon et al. 2016, the author suggests that the peptides be labeled as SmBiT and HiBiT, respectively, throughout the manuscript in order to match Promega's recent naming. In the author's understanding, SmBiT refers to the peptide 114.

Response: We agree that our descriptions on 114 and 86 are misleading. We have labeled 114 as SmBiT and 86 as HiBiT in our revised manuscript, which is consistent with the naming in Dixon et al. 2016 paper.

**Reviewer #3:**

This manuscript reports a 3.2 A cryo-EM structure of VIPR1-G protein in complex with an activating peptide PACAP27. The authors creatively included a newly developed NanoBiT method to strengthen the binding interface of VIPR1 and Gs protein, which provided an option for future structural analysis between GPCR and downstream G protein or other adaptors. The structure is solid, and resembles previous determined class B complex structures, and the structural mechanism for how peptide triggers the conformational change and receptor activation is well analyzed and described. The paper is well written too.

Response: We thank the Reviewer for the positive comment.

Main questions:

1. The ECD is surprisingly missing in recent cryo-EM structures, which looks odd as the well accepted mode suggests binding of peptide first to the ECD and then to the TMD pocket, and the helical structure of the hormone peptide is supposed to be quite rigid to stabilize the orientation of the bound ECD. Furthermore, there are literatures suggesting additional disulfide linkage between the ECD N-terminus and the ECL1 (e.g., vilardaga, 1997), which may further strengthen the orientation of ECD. The recently published PAC1 complex structure may give some clue about this by showing continued density of the ECD N-terminus and ECL1. Is it possible for the authors to revisit the original EM data of VIPR1 to see if there are more clues, or comments on this issue?

Response: We thank Reviewer’s insightful suggestions. We agree that the ECD is important for the initial recognition of the C terminus of the peptide. We indeed observed a relatively weak yet obvious density corresponding to the ECD of the receptor during image processing, including 2D class averages and 3D classifications. However, as the recently published cryo-EM structures of class B GPCR-G complexes suggested that the ECD of class B GPCR exhibits different rigidity features. For instance, the ECD of PTH1R displayed more conformational heterogeneity than that of PAC1 receptor, GLP-1R, and GCGR. We speculate that the ECD of VIP1R is somewhat extremely mobile, responsible for the averaging out of the ECD during reconstruction. Therefore, we cannot analyze the potential disulfide bond between the ECD N-terminus and the ECLs due to the relatively low local resolution. We also tried additional focused classification and refinement with the alignment on the entire receptor, unfortunately failed to sort out stable conformation of the ECD or improved the local resolution of the ECD (please see the details of the result in our responses to Reviewer #4).

2. Since the close analog of VIPR1, PAC1R, was published recently in Molecular Cell, can the author compare further with that structure? Or at least, what may be the mechanism that PACAP can recognize both receptors but VIP binds mainly with VIPR1/2?

Response: We thank the Reviewer for constructive advice. A structural comparison of PACAP-binding pockets in VIP1R and its analog PAC1R can help us to clarify the recognition mechanism of these two receptors by PACAP27. We have added the corresponding structural comparison and discussion in “PACAP recognition by VIP1R and PAC1R” section. The figures on the structural comparison of PACAP binding pocket in two receptors were added in Figure 3d-h. Additionally, we have added the discussion in the revised manuscript to provide a potential explanation of VIP selectivity for VIP1R over PAC1R (Supplementary Fig. 7).

Minor points:

1. Why use PACAP for the complex study? Was the VIP also tested for the complex formation?

Response: We have tried VIP for the VIP1R complex formation, and also can obtain a VIP-VIP1R-Gs complex. However, under our experimental conditions, the complex is not stable for the cryo-EM structure determination compared to the PACAP27-VIP1R-Gs complex.

2. It is brilliant to use the NanoBiT method for the complex formation, but it is also suspected that the NanoBiT would need.

Response: Thanks for your comment. We thought the nanobit is not a necessity for formation of the PACAP27-VIP1R-Gs complex, however, it will provide additional stabilization for the complex (Fig.1b-d, Supplementary Fig.1e) and make the complex suitable for the final structure determination under our experimental conditions. Similar stabilization effect of nanobit can be observed for CCR7/Gi complex (Supplementary Fig.3).

**Reviewer #4:**

In this paper, the authors utilised NanoBiT technology reported by Dixon et al., 2016 to enhance stability of GPCR complex for cryo-EM. For this purpose, they have chosen the peptide 86 from the manuscript, which has the highest binding affinity (Kd ~900pM). This improved the stability of two GPCR G protein complexes and could be useful for other more challenging complex. Using this approach, they have improved the biochemistry and solved the structure of VIPR-Gs complex bound to PACAP-27. Notably, this is a novel structure of a class B GPCR that has not been previously solved. Although the ECD of the receptor was poorly resolved, there are good densities for 7TM, G proteins and N-term of peptide, in accord with reported 3.2A resolution.

We thank the Reviewer for the positive comment.

Major comments:

Constructs of receptor (WT, truncated, truncated + LgBiT) were validated; however, there was no validation for Gbeta construct fused with SmBiT.

Response: We thank the Reviewer for the comment. We have validated the PACAP27-mediated activation on WT, truncated, and truncated VIP1R-LgBiT fusion, as well as Gβ-HiBiT (Supplementary Fig.1c). The truncated VIP1R exhibited a comparable response to PACAP27 compared to WT. The LgBiT fusion to truncated receptor or co-transfection of the truncated receptor with Gβ-HiBiT does not affect PACAP27-induced VIP1R activation.

Method for NanoBit assay performed for evaluating G protein coupling activities (Table S4) was not described.

Response: We apologized for our negligence. The method in Table S4 should be the TR-FRET assay which measures the cAMP accumulation, but not the NanoBiT assay which evaluates G protein coupling. It has been revised to correct description in our revised manuscript.

Methods for complex formation of CCR7/Gi, scFv16 generation were not described.

Response: We are sorry for our negligence. The methods for complex formation of CCR7/Gi is similar with the PACAP27-VIP1R-Gs complex, and we have added this method in the corresponding section in our revised manuscript.

Lack of detail in construct generated for VIPR, was there a linker between C-term of the receptor and LgBiT? It was not mentioned that there was no density observed for the LgBiT and SmBiT as they should be present in the sample. Was there any density observed for the NanoBiT in the intermediate cryo-EM map during processing?

Response: We thank the Reviewer for the comment. The flexible C-terminus of VIP1R serves as the natural linker to connect LgBiT directly. The wild type VIP1R and different lengths of C-terminus truncated VIP1R at L437, G424, and K417 were screened for assembly of the complex. When the receptor was truncated to L437, the components can be assembled into the VIP1R(31-437)-VIP1R-Gs complex with a equal proportion, suggesting a better assembly efficacy for the complex. Corresponding comments about have been added in text.

Additionally, the density of the NanoBiT is invisible in our structure. We suspect that the NanoBiT can increase the local concentration of Gs hetertrimer near VIP1R, and also make the Gs hetertrimer not easily dissociated from the receptor. Compared with the VIP1R-Gs protein complex, the NanoBiT is relatively flexible because of the existence of the flexible region between LgBiT and the receptor.

The density of receptor ECD was almost invisible in the cryo-EM map, ECD of class B receptor has a vital role in peptide binding and there have been differences in the orientation of ECD relative to the receptor core observed for different class B receptors. Has the author tried to alternative method i.e creating a mask on the receptor only to improve the ECD region?

Response: We are grateful for Reviewer’s suggestion and we have tried additional 3D classification and refinement with a mask on the entire receptor to resolve the conformational heterogeneity of the ECD. Unfortunately, neither of these trials could significantly improve the density quality of the ECD region. The following figures show the results of 3D classification and refinement with a mask on the receptor.

Fig.1 3D classification with mask on the entire receptor

Fig.2 Refinement with mask on the entire receptor

Density for the peptide appeared to be too poor to be confidently modelled after residue 15 (at least in the map supplied). Interactions beyond residue 15 in the peptide should not be discussed.

Response: We agree and have focused our discussion on the interactions from H1 to R14 of the peptide.

Minor comments:

Negative stain micrograph in Figure 1d has poor resolution, I failed to observe any differences between the two.

Response: Thank you for your comment. We have reperformed the negative stain analysis, and updated Figure 1d with a new set of micrographs to clarify the effect of NanoBiT on complex assembly.

Typo - Line 390, “barelly”

Response: Thanks for your carefully review. We have corrected the typo in our revised manuscript.

While SmBiT fused with chain B was included, LgBiT sequence fused with chain R was not included in the PDB report.

Response: Thank you for pointing out our negligence. We have updated the PDB file, in which the LgBiT sequence has been included.

Lane 79, ref 14 – this review did not discuss impact of cryo-EM on GPCR structural biology.

Response: Thank you for pointing out the incorrect reference. We have replaced the ref14 to “Illuminating GPCR signaling by Cryo-EM. [*Trends Cell Biol*.](https://www.ncbi.nlm.nih.gov/pubmed/?term=Illuminating+GPCR+signaling+by+Cryo-EM) 2018 Aug;28(8):591-594”.

**Reviewer comments, second version:**

Reviewer #1 (Remarks to the Author):

I commend the authors for having satisfactorily addressed this reviewer's comments. My only remaining suggestion is that the authors should refer to the NanoBit system as a complementation assay based on split luciferase, and not merely refer to it as a 10-stranded beta barrel protein.


Reviewer #2 (Remarks to the Author):

Most of the reviewer's concerns were now addressed by the authors' revised manuscript. Especially, the reviewer appreciates reexamination of the mutant study using CHO cell line, which offers a nice, low-background host.
The reviewer, however, would like to request the authors to fulfill two points before the manuscript is accepted. Firstly, the authors still fail to address the concern about expression levels of mutant constructs. Without experimental data showing unchanged cell-surface expression levels between WT and mutants, they cannot conclude that change in pEC50 is reflection of altered ligand binding. Secondly, the authors should describe the lack of Nanobit density and potential reasoning in the main text. This was also pointed by another reviewer (#4). Description of the unobserved density will not ruin the value of study. Rather, it will help readers understand the nature of Nanobit tethering.


Reviewer #4 (Remarks to the Author):

All of my comments/suggestions have been satisfied in the revised manuscript.

**Author rebuttal, second version:**

**Reviewer #1:**

I commend the authors for having satisfactorily addressed this reviewer's comments. My only remaining suggestion is that the authors should refer to the NanoBit system as a complementation assay based on split luciferase, and not merely refer to it as a 10-stranded beta barrel protein.

Response: We thank the Reviewer for all the constructive comments. As the Reviewer suggested, we have rewritten the sentence as follows: “NanoBiT system is one of protein-fragment complementation methods based on split luciferase, which is originally developed to monitor protein-protein interactions. The fragments of the LgBiT and SmBiT are genetically fused to a pair of interacting proteins. The interaction of fusion partners leads to structural complementation of LgBiT with SmBiT, generating a functional NanoBiT enzyme with a detectable luminescent signal”.

**Reviewer #2:**

Most of the reviewer's concerns were now addressed by the authors' revised manuscript. Especially, the reviewer appreciates reexamination of the mutant study using CHO cell line, which offers a nice, low-background host.

The reviewer, however, would like to request the authors to fulfill two points before the manuscript is accepted. Firstly, the authors still fail to address the concern about expression levels of mutant constructs. Without experimental data showing unchanged cell-surface expression levels between WT and mutants, they cannot conclude that change in pEC50 is reflection of altered ligand binding. Secondly, the authors should describe the lack of Nanobit density and potential reasoning in the main text. This was also pointed by another reviewer (#4). Description of the unobserved density will not ruin the value of study. Rather, it will help readers understand the nature of Nanobit tethering.

Response: We thank the Reviewer for the constructive comment. We have assessed the cell surface expression of VIP1R mutants by cell cytometry. The detection method for cell surface expression and the corresponding expression data have been added in the revised manuscript (Fig.S1C and Table S4).

Additionally, we have added the description of the lack of NanoBiT density and the putative reason in the main text as follows: “Interestingly, the density of the NanoBiT is invisible in our structure. We suspect that the NanoBiT can increase the local concentration of Gs hetertrimer near VIP1R, and also make the Gs hetertrimer not easily dissociated from the receptor. Compared with the VIP1R-Gs protein complex, the NanoBiT is relatively flexible because of the existence of the linker between LgBiT and the receptor. The stabilization mode of NanoBiT is different from the antibodies which bind against the antigen directly and can be traced in a clear density map.”

**Reviewer #4:**

All of my comments/suggestions have been satisfied in the revised manuscript.

Response: We would like to thank the Reviewer for all the constructive comments on our manuscript.

**Reviewer comments, third version:**

Reviewer #2 (Remarks to the Author):

Both of the reviewer's points were satisfactorily addressed by the authors' revised manuscript.

**Author rebuttal, third version:**
